# Esh-Shaheinab: The archetype of the Sudanese Neolithic, its premises and sequels

**DOI:** 10.1371/journal.pone.0309600

**Published:** 2024-10-31

**Authors:** Giulia D’Ercole, Julie Dunne, Giacomo Eramo, Richard P. Evershed, Elena A. A. Garcea

**Affiliations:** 1 Institut für Ägyptologie und Koptologie, Egyptian Archaeology, Ludwig-Maximilians-Universität München, Munich, Germany; 2 Organic Geochemistry Unit, School of Chemistry, University of Bristol, Bristol, United Kingdom; 3 Dipartimento di Scienze della Terra e Geoambientali, Università di Bari Aldo Moro, Bari, Italy; 4 Dipartimento di Lettere e Filosofia, Università di Cassino e del Lazio Meridionale, Cassino, Italy; University of California Santa Cruz, UNITED STATES OF AMERICA

## Abstract

Esh-Shaheinab is a landmark in the African Neolithic. This site gave the name *Shaheinab Neolithic* to the Neolithic period in central Sudan, becoming its archetype. Excavated in the late 1940s by A.J. Arkell, it bears witness to the processes of domestic animal introduction from the Middle East into North and East Africa. Its excavation also uncovered the remains of an earlier Mesolithic or *Early Khartoum* (ca. ninth-sixth millennia BC) and a Late Neolithic occupation (ca. fourth millennium BC), providing essential insights into the Neolithic’s premises and sequels. Although the influence of Esh-Shaheinab has been recognized for more than seventy years, our knowledge of its material culture has remained as it was then. In 2001, one of the present authors (EAAG) had permission to restudy the ceramic collection at the National Museum in Khartoum and subsequently export samples for laboratory analyses. Here, for the first time, we provide a multi-scale analysis of the Esh-Shaheinab ceramic material from the Early Khartoum to the Late Neolithic periods by integrating the chaîne opératoire approach into the local landscape. By combining the results of macroscopic and microscopic analyses, we performed petrographic investigations on the composition and manufacturing technology of the ceramic pastes using polarized optical microscopy (POM) and scanning electron microscopy (SEM-EDS). Organic residue analysis (ORA) was also carried out, to provide information on diet, vessel use, and subsistence practices. The results of our combined analyses showed that the inhabitants of Esh-Shaheinab developed an adaptation specific to the ecological niche they inhabited. They lived in the western valley of the Nile, which was narrower and offered different environmental conditions than the eastern bank. This resulted in partial continuity in manufacturing traditions and ceramic recipes, including more mixed wadi materials and a strong emphasis on wild meat consumption as the narrower alluvial plain restricted animal herding.

## Introduction

**Fig 1:**
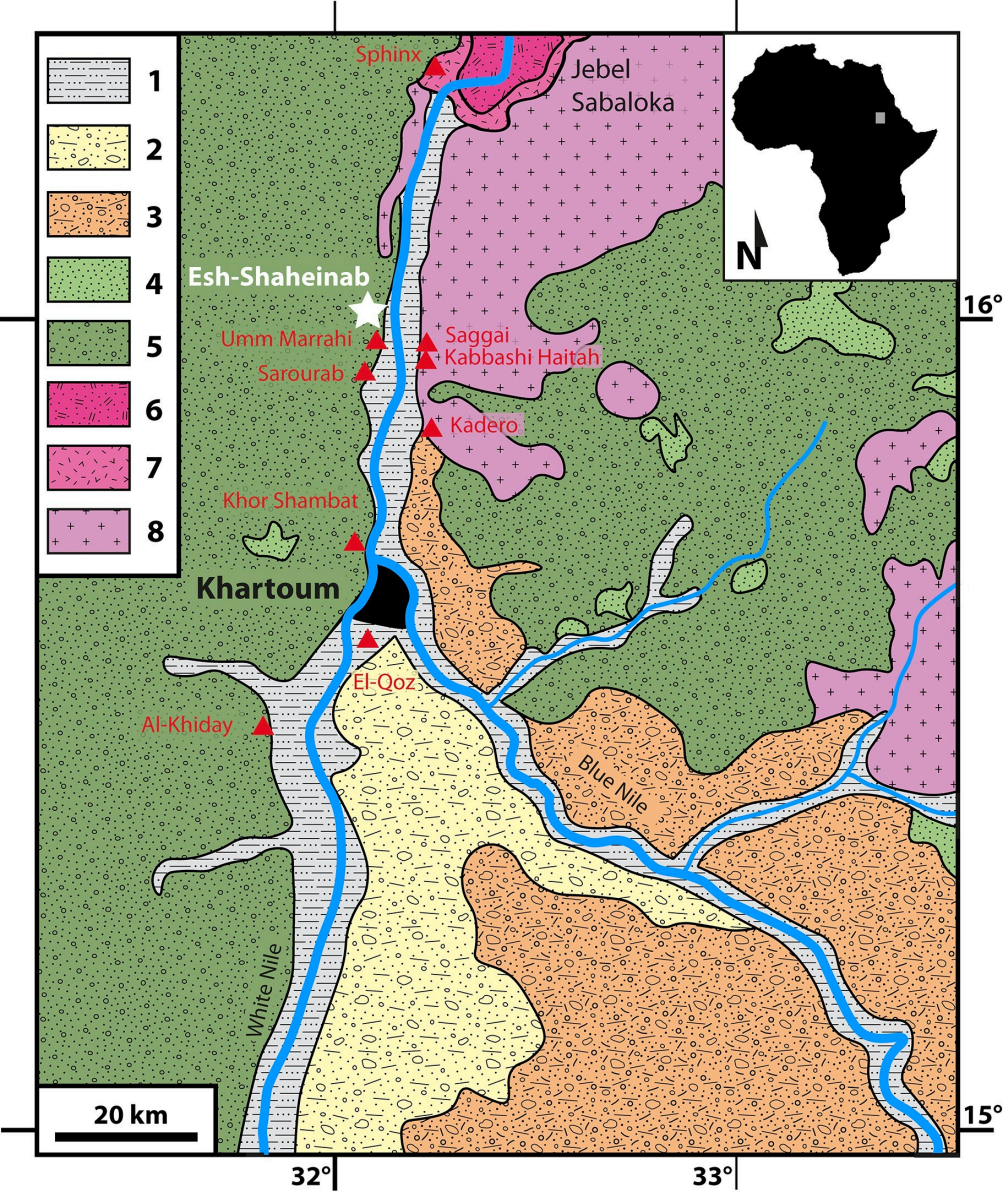
Schematic geological map of the Khartoum area and location of the sites cited in the text redrawn from El Tahir et al. (2013) [66] and D’Ercole et al. 2017 [67] using Adobe Illustrator 2023. Key: 1 = recent alluvium; 2 = older alluvium; 3 = Gezira Formation; 4 = Umm Badda member (Omdurman Formation); 5 = Merkhiyat member (Omdurman Formation); 6 = Younger granites; 7 = Granitic ring dyke; 8 = Precambrian Basement Complex. See text for details.

The archaeological site of Esh-Shaheinab, located in the western Middle Nile Valley in central Sudan (Fig 1), set the foundations of the Sudanese Neolithic in the mid-1900s, becoming the archetype of this cultural phase. It was chosen for excavation on behalf of the then Sudan Antiquities Service in 1949 by A.J. Arkell, the first to be appointed as Commissioner for Archaeology and Anthropology for the Sudan within the National Museum of Antiquities in Khartoum, who led the excavations [1]. The site is primarily known for its Neolithic settlement, although excavations brought to light remnants of a previous Early Khartoum (Mesolithic) occupation and later, Late Neolithic (fourth millennium BCE) and Meroitic (280 BCE-CE 400) graves.

Since then, the *Shaheinab Neolithic* has been a renowned term of reference for African and Middle Eastern prehistory. Numerous other sites later contributed to characterize and date what is now widely known as the Shaheinab Neolithic in central Sudan, which spans the period between 5000 and 3800 BCE, corroborating the Shaheinab chronology [2]. Arkell [1] obtained the first two radiocarbon dates in the 1940s, thanks to a collaboration with the founder of the radiocarbon dating method, W.F. Libby, when this method was at its beginnings. Charcoal from a hearth in the Neolithic occupation was then dated to 5060±450 uncal BP (C-753) or 4988–2765 cal BCE (OxCal 4.4, 95.4%), and shell was dated at 5446±380 uncal BP (C-754) or 5288–3516 cal BCE. The site was re-excavated in 1979–1980 and provided three other radiocarbon dates on shell for the Neolithic settlement [3]. They are 5550±90 uncal BP (T-3699) or 4611–4171 cal BCE (median 4391 cal BCE), 5650±60 uncal BP (T-3222) or 4656–4352 cal BCE (median 4504 cal BCE), and 5720±80 uncal BP (T-3223) or 4774–4363 cal BCE (median 4568 cal BC), placing the timespan of the Neolithic occupation across the mid-fifth millennium BCE (4700–4200 BCE). The Early Khartoum levels at Esh-Shaheinab have not been radiometrically dated. However, Arkell [1] assigned them to the ‘Dotted Wavy Line horizon’, which since then has been considered as a late phase of the Early Khartoum period (see below). The overall Early Khartoum period in central Sudan is currently dated between 8800–8500 and 5000 BCE [2].

Although Esh-Shaheinab is a landmark in the African Neolithic, particularly for its related processes of domestic animal acquisition and for the spread of a food producing economy from the Middle East, its material culture has never been studied in detail either from a technological, chronological, or critical point of view. In 2001, one of the authors of this paper (EAAG) was granted permission by the Sudan National Museum to access the ceramic collection from Arkell’s excavations kept in the museum storage in Khartoum. During her stay in the museum, she was able to conduct macroscopic analyses and geostatistical mapping of the ceramic assemblage according to up-to-date criteria based on the reconstruction of the manufacturing sequences of Early Khartoum, Neolithic, and Late Neolithic ceramic productions and site organization during these three periods [4–6]. Permissions were also granted to export samples for petrographic, mineralogical, and geochemical analyses.

This paper offers, for the first time, a multi-scale analysis of the Esh-Shaheinab ceramic material from the Early Khartoum to the Neolithic and Late Neolithic periods and integrates the chaîne opératoire approach on ceramic assemblages within the landscape. By combining the results of macroscopic and microscopic analyses, we provide an essential missing piece to the outstanding value of Esh-Shaheinab for Holocene prehistory in Africa, the Mediterranean, and the Middle East, with its premises in the Early Khartoum period and sequels in the Late Neolithic. In particular, we describe and discuss the petrographic investigations on the composition and manufacturing technology of ceramic pastes using polarized optical microscopy (POM) and scanning electron microscopy (SEM-EDS). Furthermore, lipid residue analysis provides valuable information on diet, vessel use, and subsistence practices at the site across several millennia, from the Early Khartoum (ca. ninth-sixth millennia BCE) to the Late Neolithic (ca. fourth millennium BCE) periods.

## Environment, geomorphology, and economy at Esh-Shaheinab

Esh-Shaheinab is located on the Early Holocene western (left) bank of the Middle Nile about 30 km south of the Sixth Nile Cataract (Jebel Sabaloka) and 50 km north of Omdurman, the sister city of Sudan’s capital (Khartoum) at the confluence of the White Nile and the Blue Nile, in central Sudan (Fig 1). The archaeological site is about 200 m long and 50 m wide and extends on a gravel ridge of water-worn quartz pebbles eroded out of the Nubian Sandstone Formation and transported by a small Nile tributary, wadi Abu Shush.

The alluvial sediments downstream of the confluence of the White Nile and Blue Nile mainly consist of contributions from the Precambrian magmatic-metamorphic basement and Mesozoic sandstones. The Precambrian Basement Complex consists of igneous and metamorphic rocks [7, 8], especially granites and gneisses, as part of the Mozambique Belt (Pan-African orogeny), which are well exposed on the eastern side of the River Nile in the Khartoum area (Fig 1). At about 30 kilometers north of Esh-Shaheinab, the magmatic complex of the Jebel Sabaloka (Lower to Middle Devonian) is characterized by several cycles of intrusive and effusive activity coupled with cycles of subsidence [9].

The Nubian Sandstone Formation outcrops across much of the investigated area (Fig 1). The Omdurman Formation, with the two members Umm Badda (Late Albian-Turonian) and Merkhiyat (Turonian-Senonian?), attests to a continental paleoenvironment that gradually shifts from a fluviolacustrine to a braided river environment [10–13]. The dominant lithologies are shale, siltstone and sandstone, with pebbly sandstones and conglomerates being rare [13].

The Gezira Formation (Oligocene-Quaternary) covers the area between the White Nile and the Blue Nile, lying unconformably on either the Nubian Sandstone Formation or directly on the Basement Complex [14, 15]. It consists mainly of unconsolidated gravels, sands, and, less frequently, silt and clays. The increasing smectite/kaolinite ratio from the lower to the upper part of the Gezira Formation points to an almost tropical climate, which shifted toward a period of increasing aridity and became relatively wet during the Holocene [13]. Quaternary deposits in the area include the Nile silt and wadi deposits.

A layer of small and larger pebbles, seemingly deposited in Acheulean times (ca. 800,000–200,000 BP), overlies the Nubian Sandstone bedrock, and a 2 m thick deposit of black alluvial clay was superimposed on the Early Stone Age (Acheulean) gravel. The gravel ridge with the Holocene archaeological site lays at the top of this deposit. This gravel began to accumulate during the Early Khartoum period when the site was probably an island [1].

Arkell [1] suggested that flood levels were about 10 m higher during the Early Khartoum period than at present, reducing to +5 m during the Neolithic period. He also proposed that the Early Khartoum occupation below the Neolithic levels took place in the dry seasons, during low-river levels. Changes in the Nile levels involved different faunal spectra, with swamps in the Early Khartoum, where kob and marsh cane rat lived [16]. Subsistence was based on plant and mollusc gathering, fishing, and hunting in riverine or lacustrine environments [2].

During the Neolithic, more drought-tolerant species, such as hare, kudu, antelope and gazelle, appeared, suggesting a shift to forest conditions by the river and savanna inland. Giraffe and hippopotamus were the most plentiful wild species at this time. The presence of these and other large animals (e.g., elephant, rhinoceros) hinted at more successful hunting methods in the Neolithic [1]. Fish remains were common, although less frequent than in the Early Khartoum period. At first sight, domestic species seemed to account for only 2%, including dwarf goat, sheep or goat, and sheep, with no remains of cattle [1]. However, a review of the faunal remains indicated that no osteological arguments could support the existence of two different goat breeds [17], as previously assumed, and that bones previously identified as wild buffalo were, in fact, domestic cattle [16, 18]. The reanalysis of the faunal remains still confirmed a prevalence of wild species (84%, [16]) but demonstrated a higher frequency of domestic animals (16%) in the economic spectrum, supporting a mixed hunting-fishing-gathering and herding economy. A few remains of domestic dogs were also found at Esh-Shaheinab [16, 17]. No remains of domestic plants occurred at the site, although numerous *Celtis* seeds revealed that wild fruits were gathered for food [19].

The low frequency of domestic species may reflect a gradual introduction of livestock, exploitation for purposes other than meat, or restricted killing. Domestic species accounted for a prevalence of ovicaprids (75%) over cattle (25%) ([16]; S1 Table). Considering the geomorphological features of the western Nile bank, which is narrower than the eastern one and displays hilly slopes and restricted alluvial soils, offering limited grazing opportunities, may have affected herding practices for the early pastoralists at Esh-Shaheinab [17].

No herbivore dung was recorded in the pottery of any period from Esh-Shaheinab. Interestingly, the use of herbivore dung is mainly related to pottery assemblages from sites with a faunal record and overall economy dominated by ovicaprids, such as for example, the A-Group and Pre-Kerma sites in northern Sudan, and the sites of the Handessi culture in the Wadi Howar [20, 21]. Conversely, at Esh-Shaheinab domestic caprines only represent 25% in the rather scarce domestic fauna [16].

## Early Khartoum and Shaheinab Neolithic

Arkell [22] first used artefactual materials to distinguish between what he initially called *Wavy Line Culture* (Mesolithic) and *Gouge Culture* (Neolithic), respectively, taking a ceramic decoration made with incised wavy line motifs, and a lithic implement, flaked and polished celts (gouges), as discriminating archaeological hallmarks. He soon changed the terms into *Khartoum Mesolithic* or, preferably, *Early Khartoum*, and *Khartoum Neolithic* or, preferably, *Shaheinab Neolithic* to specify the economic organization and relative chronology of these two cultures [1]. His excavations at a nearby site, El-Qoz [1], confirmed his observations from Esh-Shaheinab, that is, a stratigraphic sequence of occupational levels associated with earlier hunter-fisher-gatherers (Early Khartoum) and later food producers (Shaheinab Neolithic). Furthermore, Arkell identified Dotted Wavy Line patterns as a ‘typological link between the pottery of the Khartoum Mesolithic and the pottery of the Khartoum Neolithic’ ([1]: 69).

In general, the Early Khartoum complex includes high artifact densities and expansive sites, covering between 1 and 4.5 hectares. The material culture consists of a microlithic industry, mostly on quartz, systematic production of decorated (incised and impressed) pottery, ground stones, bone tools, and ostrich eggshell beads [2].

Several technological and economic (food production) innovations appeared at Esh-Shaheinab and characterized the Neolithic period of the fifth millennium BC in central Sudan [2]. Barbed bone harpoons, shell (*Aspatharia* and *Etheria*) fishhooks, stone and bone borers, amazonite beads, zeolite and quartz crystal lip-plugs, burnished pottery, black-topped red ware and black ware, gouges, and rare mace-heads were the major novelties recorded at this site. Ornaments of shells of land and freshwater molluscs were also worn for personal decoration. Finally, Arkell [1] suggested that rhyolite gouges were not manufactured on the spot, while bone celts were. To date, the only known sources of rhyolite are in the volcanic outcrops on Jebel Sabaloka at the Sixth Cataract of the Nile [23] (Fig 1). Compared to Early Khartoum stone tool-kits, lunates were more frequent, scrapers were better finished, borers were more numerous and diversified, and grinders were more standardized. Hearths were numerous and the only household features in the Neolithic levels.

## The ceramic assemblage: Macroscopic data

The ceramic assemblage from Arkell’s excavations kept at the Sudan National Museum in Khartoum consists of 969 potsherds [4, 5]. It includes a majority of Neolithic sherds (764 pieces: 78.8%), with a relative quantity of Early Khartoum (177 pieces: 18.3%), and a few Late Neolithic (28 pieces: 2.9%) specimens.

**Fig 2:**
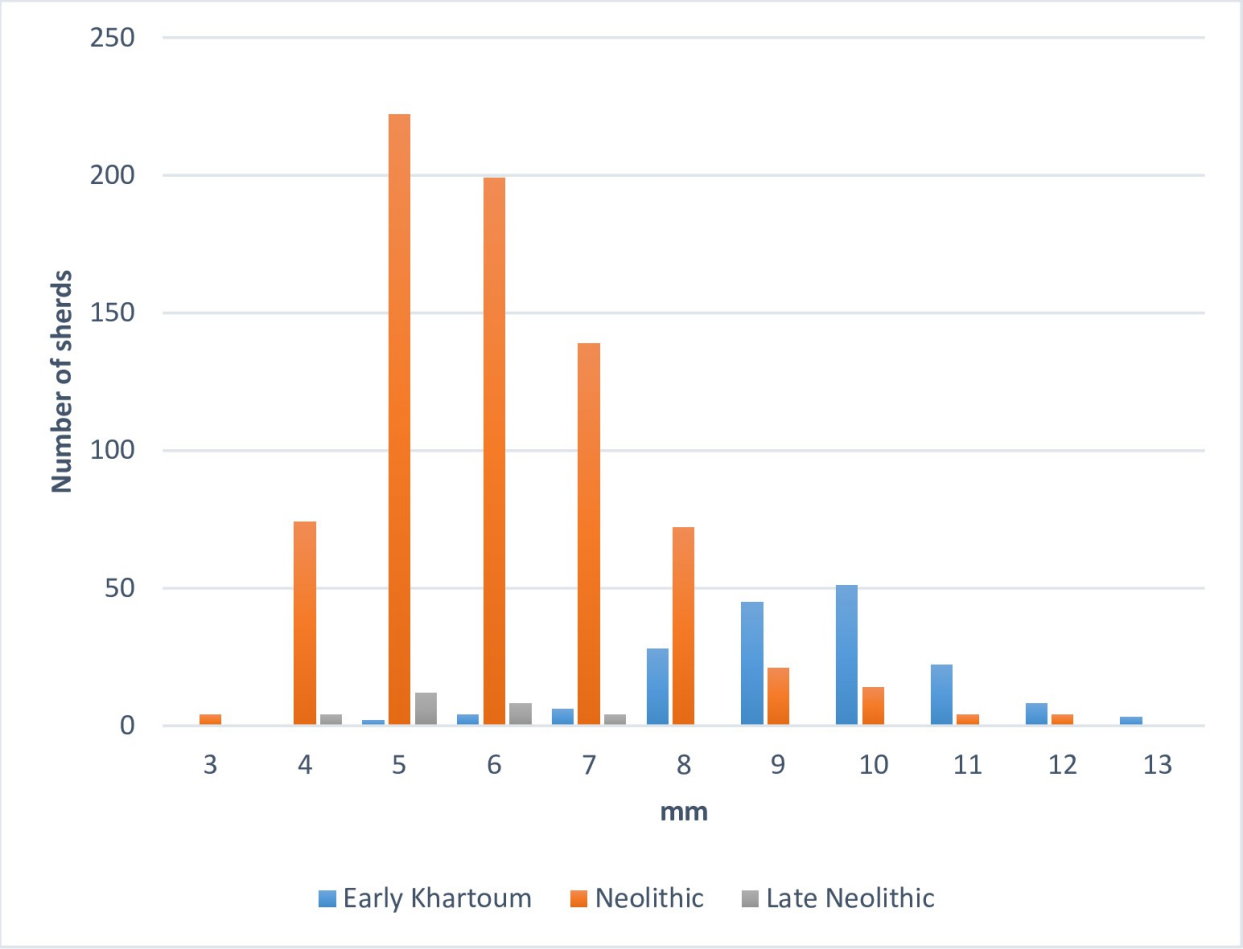
Wall thicknesses of Early Khartoum, Neolithic, and Late Neolithic sherds.

Vessels have quite different wall thicknesses in the various periods. Early Khartoum vessels are the thickest (5–13 mm), with a marked mode around 10 mm. Neolithic (3–12 mm) and Late Neolithic (4–7 mm) sherds indicate a mode around 5 mm [4], although Late Neolithic vessels are never thicker than 7 mm (Fig 2).

**Fig 3:**
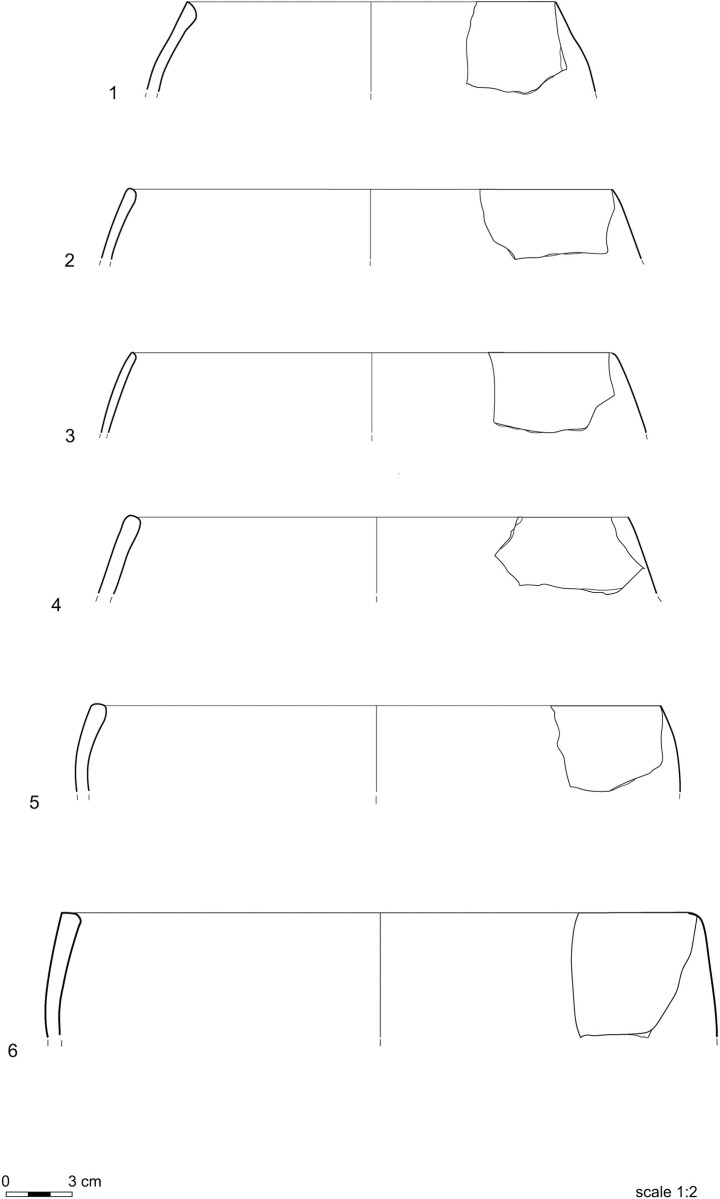
Ovoid and globular vessels.

Information on vessel shapes is only available for the Neolithic assemblage, as the reconstructable shapes of the pots from the other periods are too few for statistical analysis. Ovoid (9–42 cm), everted (13–38 cm), and straight-walled vessels (7–39 cm) have variable diameters, whereas globular shapes (18–38 cm) are the most standardized. Ovoid and globular vessels (Fig 3) are prevalent, straight-walled types (Fig 4.1-4.3) are common, while everted shapes (Fig 4.4) seem to be rarer. The assortment of shapes of the Neolithic vessels may have been a response to diversified functional requirements at this time [4].

**Fig 4:**
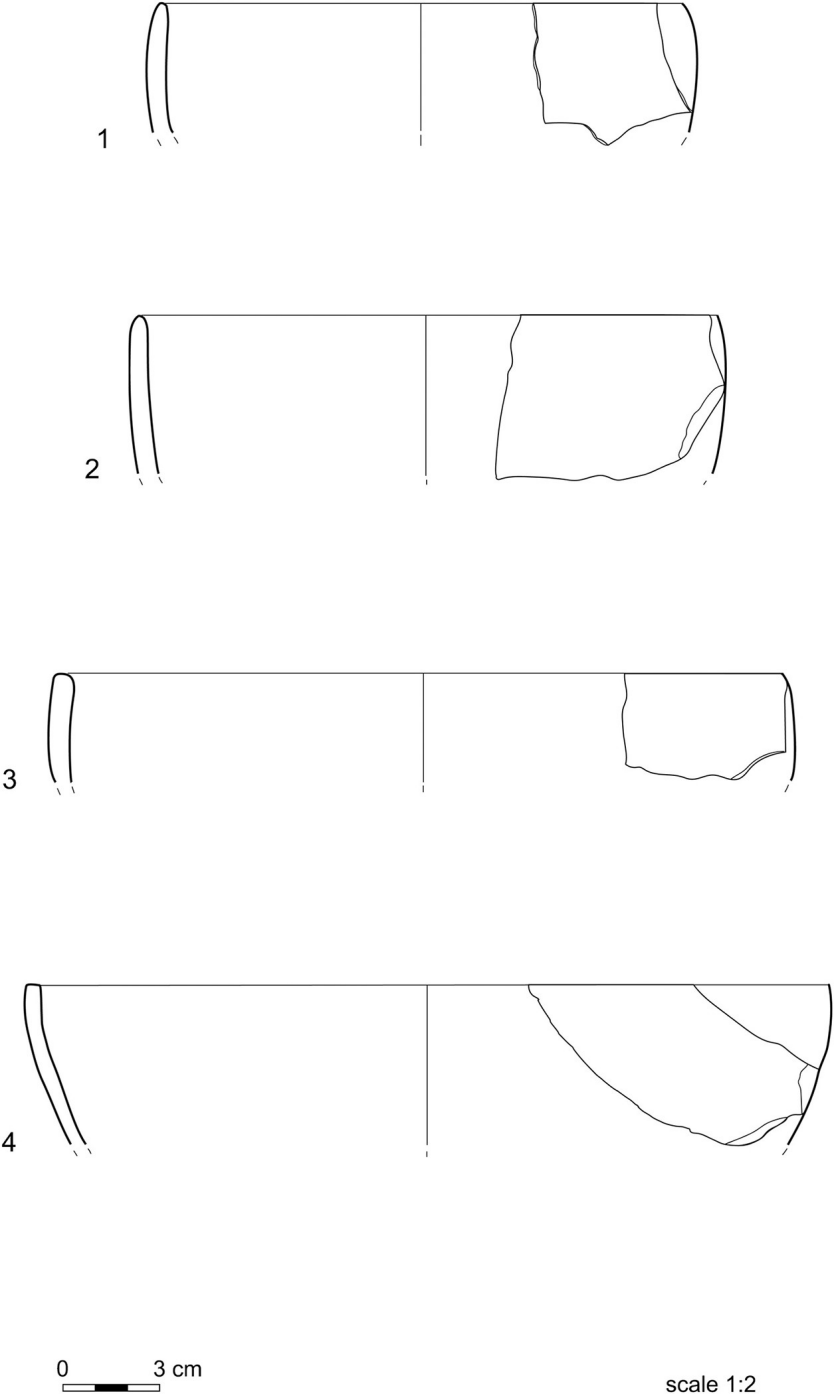
Straight-walled and everted vessels.

**Fig 5:**
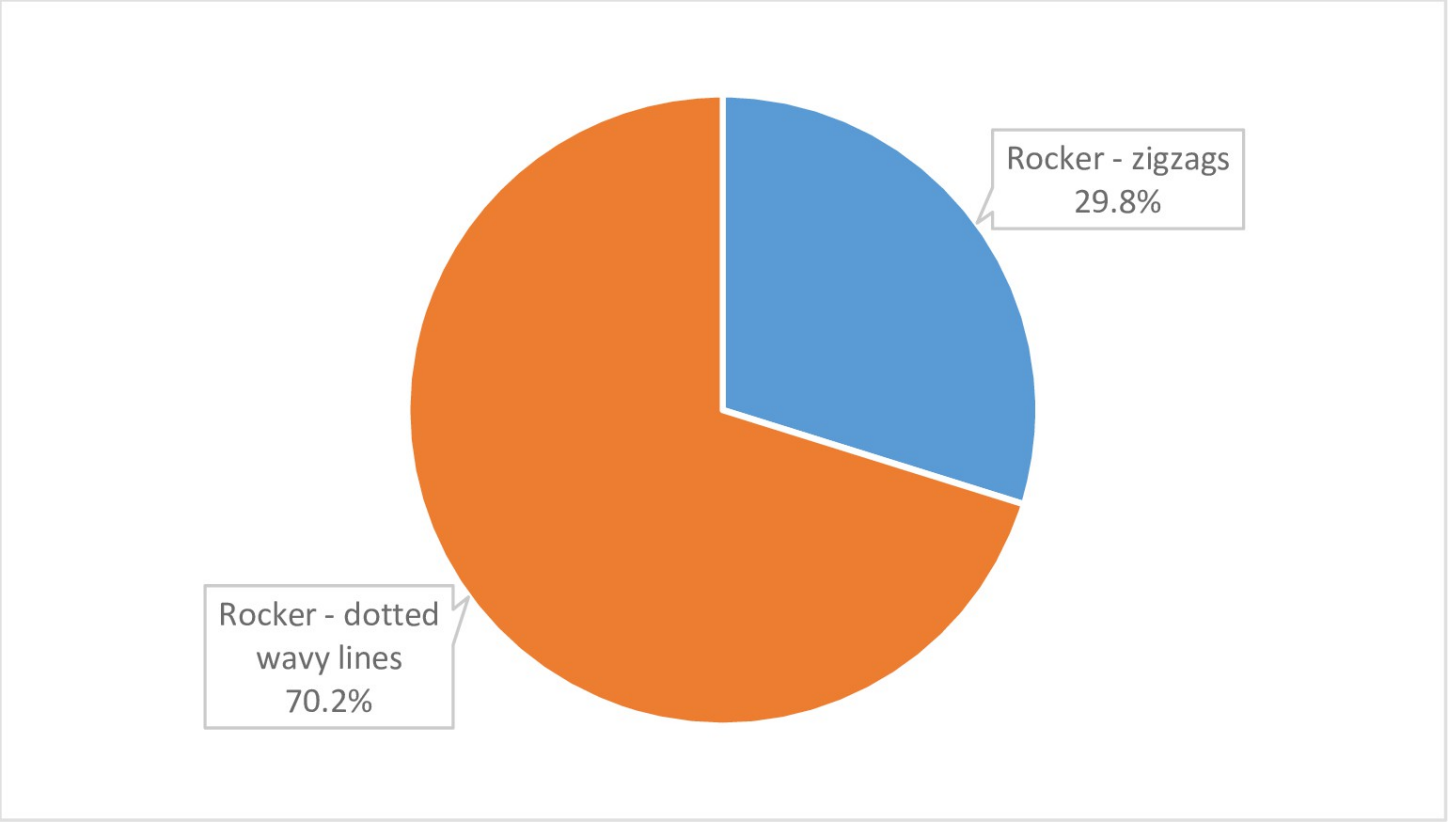
Decorated Early Khartoum pottery: Distribution of motifs within the rocker technique.

Undecorated pottery is a rarity in the Early Khartoum assemblage (3.4%), and the rest is entirely decorated with the rocker technique (96.6%), forming either dotted wavy lines or zigzags. The prevalence of dotted wavy line motifs (70.2%) of the total decorated sample (Fig 5) and the lack of incised pottery, including wavy or straight-line motifs, represents an indicator of the recent phases of the Early Khartoum period, initially referred to as the Dotted Wavy Line horizon. Some Early Khartoum sherds were modified into small discs, not necessarily during the same Early Khartoum period.

**Fig 6:**
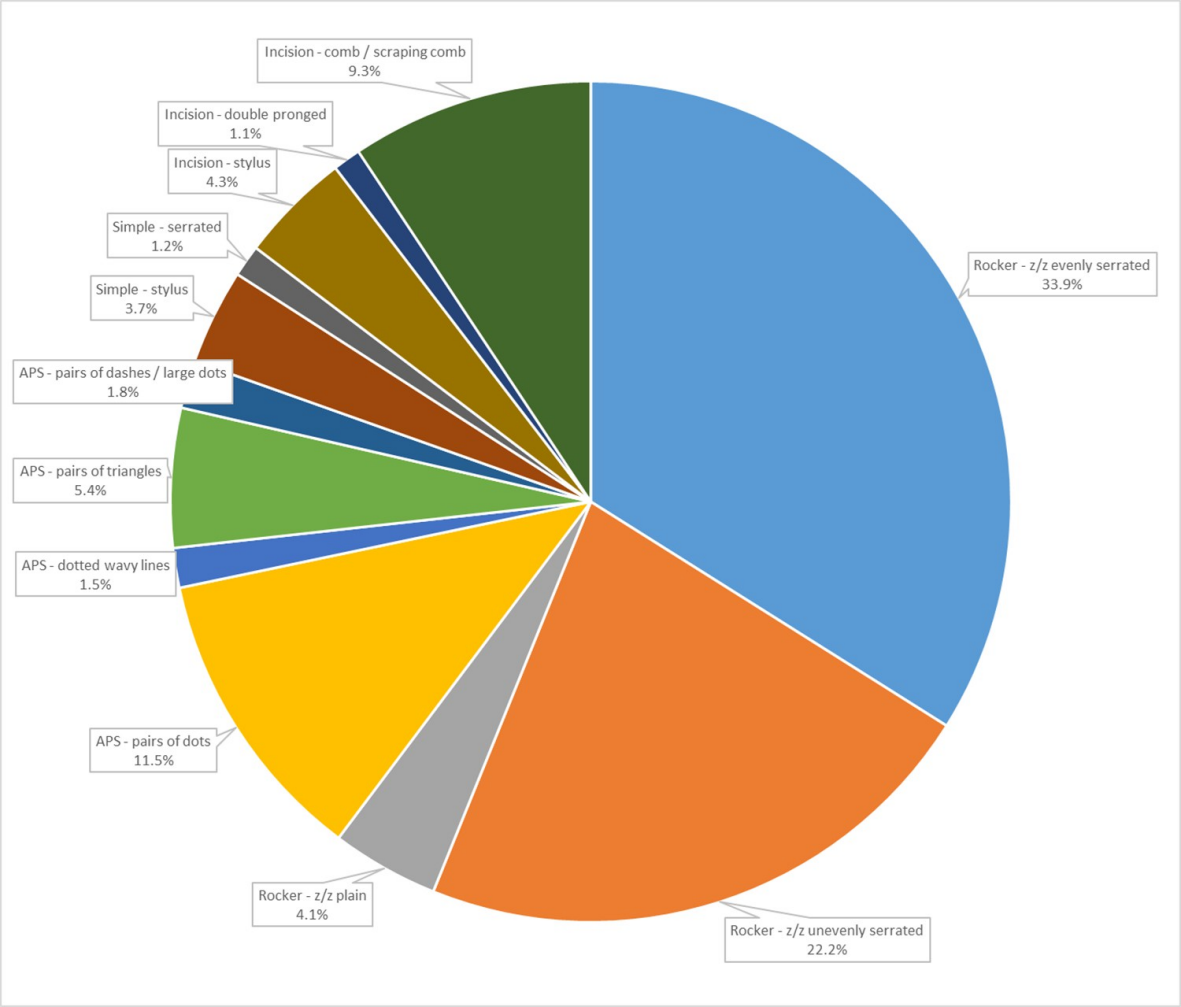
Decorated Neolithic pottery: Distribution of techniques and implements.

Neolithic pottery shows a very large variability in surface decorations and treatments, with over 60 different decoration types, suggesting the rise of a large variety of stylistic expressions at this time [4]. Undecorated sherds are 14.4%, and decorated ones are 85.6%. The variety of decorative implements is the most emblematic Neolithic feature. Of the total decorated pottery, decorative techniques include rocker (60.2%), alternately pivoting stamp (20.2%), incision (14.7%), and simple impression (4.9%) (Fig 6). Different implements could have been used in the same technique, such as combs with evenly serrated (33.9%), unevenly serrated (22.2%), or plain edges (4.1%) in the rocker technique. Concurrently, the same implements could also be used with different techniques, for example, combs with evenly serrated edges, which are used in both the rocker and the simple impression technique. Continuous zigzags of vees and dots made with an unevenly serrated comb (22.2%), and pairs of triangles (5.4%) and dots (11.5%) made with the alternately pivoting stamp technique are amongst the typical features of Shaheinab Neolithic ceramic assemblages. Paired lines made with the alternately pivoting stamp technique are organized in different motifs and do not always run parallel to the rim but can be diagonal, panelled, or arch-shaped. This technique is also used to make an alternative design to the dotted wavy line motif (1.5%) in the Neolithic period.

**Fig 7:**
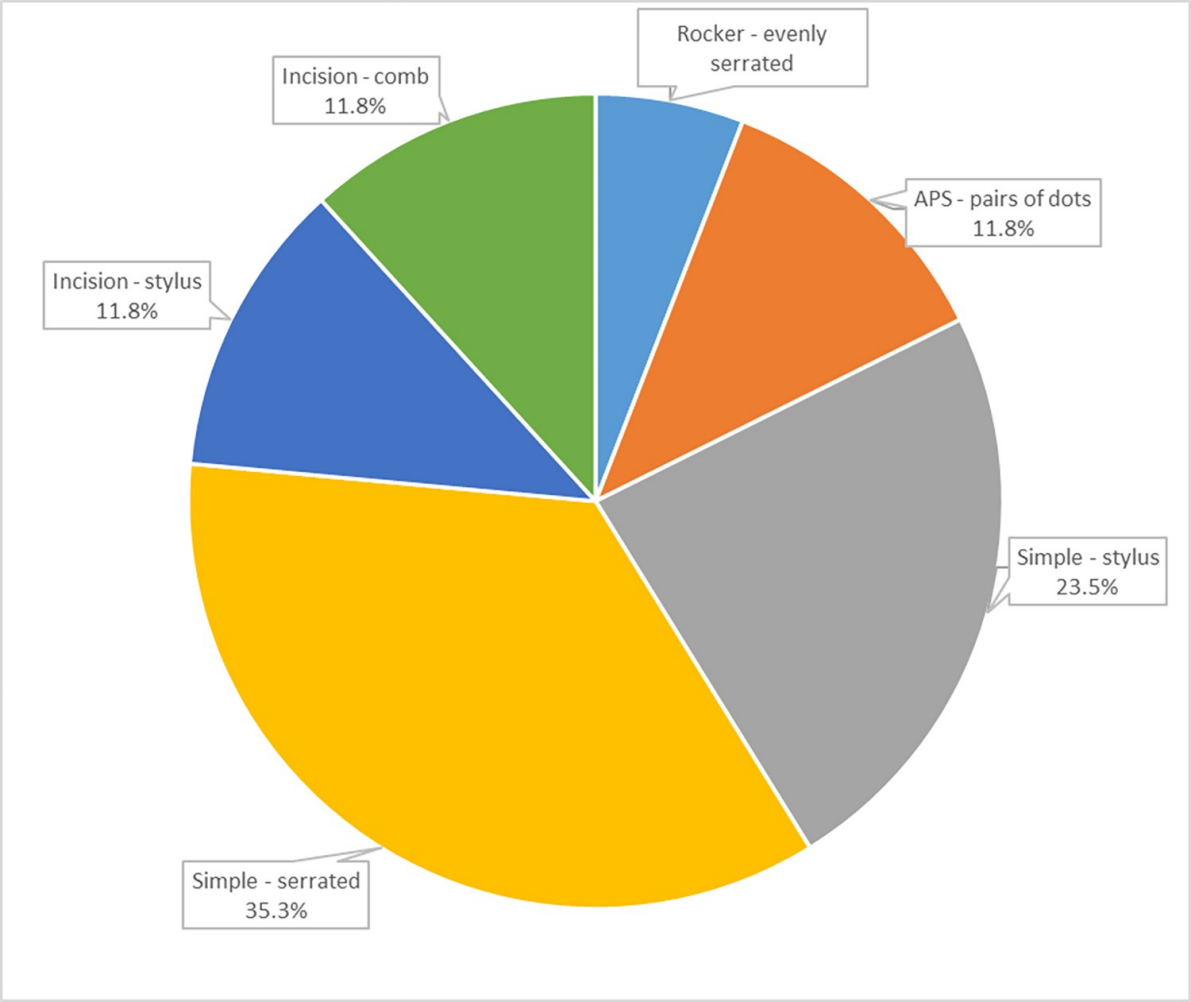
Decorated Late Neolithic pottery: Distribution of techniques and implements.

The Late Neolithic features a large amount of undecorated pottery (39.3%). The remaining assemblage is decorated (60.7%). While the variety of decorative techniques does not change with respect to the Neolithic assemblage, the types of implements and motifs consistently decrease. Zigzags with evenly serrated dots (5.9%) and paired dots (11.8%) are the only rocker and alternately pivoting stamp types, respectively. Simple impressions largely prevail, employing both stylus (23.5%) and serrated (35.3%) implements. The same is true concerning incisions, which are also made with a stylus (11.8%) and a comb (11.8%) (Fig 7). Burnishing is a common surface treatment in Neolithic and particularly Late Neolithic times [4]. Some pots are black-topped, either with a row of inverted triangles or with a band parallel to the rim. Others are red-brown or black. Black burnishing is most frequent in the Late Neolithic assemblage [4].

Thanks to Arkell’s accurate labelling of the squares and layers he identified, statistical and geostatistical analyses of intra-site pottery distribution could be conducted in order to observe spatial patterns of pottery abundance in the Early Khartoum, Neolithic, and Late Neolithic [5, 6]. Horizontal variations of pottery density were observed by plotting ceramic finds on the original contour map of the site ([1], Plate 2). Vertical patterns considered three main stratigraphic clusters (upper layers: 0/-30 cm; middle layers: -30/-60 cm; and lower layers: below 60 cm). Horizontal and vertical mapping used kriging interpolation between sampled points to make estimates of objective isopleths.

The Early Khartoum material shows a wide distribution, suggesting that this period’s occupation was more consistent than ephemeral ‘traces of a possible short occupation by a few people’ originally indicated by Arkell ([1]: 3). He assigned this occupation to a small (low river) fishing camp established before the Neolithic people settled on it. In situ Early Khartoum material occurred in respectable quantities throughout the site, and plotting confirmed the presence of undisturbed Early Khartoum layers below the Neolithic stratigraphy, particularly in squares I60/60+ and M83/70-100, at a depth of over 60 cm. Kriging analysis demonstrated that the Early Khartoum pottery was mostly concentrated in the central area of the site and spread to the eastern part [5].

The Neolithic occupation had the largest horizontal extension and was not very thick, indicating a seasonal but repetitive use of the site. The pattern of abundance of Neolithic pottery in the three stratigraphic clusters (upper, middle, and lower layers) indicated that the site occupied a larger area in this period and that the majority of the sherds laid in the upper and middle layers.

Finally, the Late Neolithic site, which was used as a burial ground, was the most ephemeral and was seemingly only visited for funerary purposes by nomadic pastoralists. Late Neolithic pottery was mostly located in the upper layer, and contour maps suggested that the very few sherds in the middle layer likely penetrated from the upper layer.

## The ceramic assemblage: Microscopic data

Although Esh-Shaheinab is the archetype site of the Sudanese Neolithic, information on the manufacturing processes of the ceramic assemblages is poor and outdated. Nordström [24] and Francaviglia and Palmieri [25] carried out initial petrographic analyses on a few Esh-Shaheinab potsherds. Considering the high frequency of silt and sand in Neolithic ceramic assemblages from central Sudan, Nordström [24] suggested a prevalent use of local raw materials (see also [26]). Previous granulometric analyses showed that these Neolithic ceramics have fine and medium-coarse matrixes [24, 26] and subangular and subrounded mineral tempers [26, 27]. The mineralogical composition hinted at a lithological provenance from the Basement Complex [27]. Quartz appeared to be the most common mineral inclusion, but feldspar and mica were also used.

Furthermore, Francaviglia and Palmieri [25] noted that the Esh-Shaheinab Neolithic pottery contains a more significant internal variability than contemporary assemblages from the sites on the eastern (right) Nile bank. They attributed this difference to the narrower floodplain on the western bank, which contains more mixed wadi and Nile sediments.

Given the importance of the Esh-Shaheinab pottery as a reference for the Neolithic in Sudan and Africa, it was imperative to systematically analyze the Esh-Shaheinab assemblages from all periods with state-of-the-art and rigorous techniques in order to reconstruct the various stages of the manufacturing sequence, including raw material procurement, processing, forming, firing and use of the vessels. The results are presented below.

## Materials

**Fig 8:**
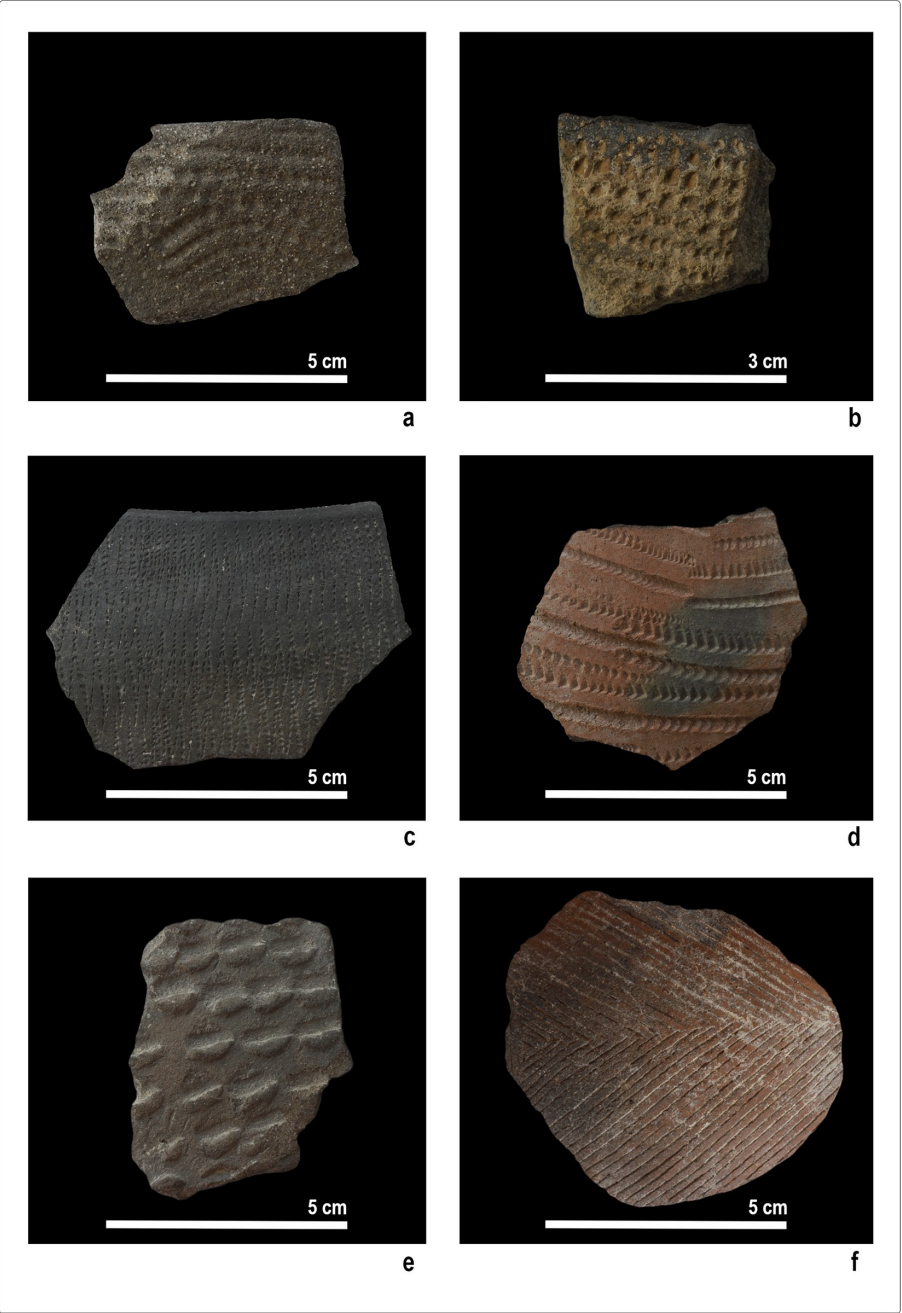
Examples of analysed samples from the various periods: a-b. Early Khartoum pottery: a. Rocker dotted wavy lines; b. Rocker packed zigzags; c-d. Neolithic pottery: c. Rocker packed zigzags with stitches; d. Rocker zigzags of “vees” and dots; e-f. Late Neolithic pottery: e. Simple impressions; f. Incisions (Photos by G. D’Ercole).

Forty ceramic samples were selected for polarized optical microscopy (POM) analysis. They came from different squares and archaeological levels of the site, aiming at representing the internal variability of the pottery corpus in terms of chronology, technology of production, and style. They comprise fifteen Early Khartoum, twenty Neolithic, and five Late Neolithic potsherds, including rims and walls, and display different thicknesses, surface treatments, and decorations (Table 1 and Fig 8). The prevalence of Neolithic sherds over those dated to the Early Khartoum and Late Neolithic periods reflects that most of the ceramic assemblage dates to the Neolithic (78.8%) (see above).

**Table 1:** Chronology, provenance, body portion, thickness, and decorative motif of samples submitted to POM, SEM, and ORA analysis.

No. Sample	Period	Sq. y axis	Sq. x axis	Layer	Body part	Body thickness (mm)	Decorative motif	Analysis
SHA 022	Early Khartoum	L	66	D	wall	9	DWL combined	POM, ORA
SHA 023	Early Khartoum	L	66	D	wall	10	DWL	POM, ORA
SHA 024	Early Khartoum	J	60	30–50	wall	7	DWL combined	POM, ORA
SHA 025	Early Khartoum	I	59	1–20	wall	8	DWL combined	POM, SEM, ORA
SHA 026	Early Khartoum	L	59	(8) 30–50	wall	9	DWL combined	POM, ORA
SHA 050	Early Khartoum	J	59	D	wall	9	DWL combined	POM, SEM, ORA
SHA 051	Early Khartoum	G	59	S	wall	10	Rocker packed zigzags	POM, ORA
SHA 052	Early Khartoum	G	59	S	wall	8	DWL	POM, ORA
SHA 053	Early Khartoum	M	66	D	wall	12	DWL combined	POM, SEM, ORA
SHA 054	Early Khartoum	I	60	10–40	wall	6	DWL	POM, ORA
SHA 055	Early Khartoum	N	88	20–40	rim	8	DWL + milled impressions	POM, ORA
SHA 056	Early Khartoum	H	59	20–40	wall	7	Rocker packed zigzags	POM, SEM, ORA
SHA 057	Early Khartoum	H	59	20–40	wall	9	Rocker packed zigzags	POM, ORA
SHA 058	Early Khartoum	J	59	40–60	wall	7	DWL	POM, ORA
SHA 059	Early Khartoum	N	87	40–60	wall	7	Rocker packed zigzags	POM, ORA
SHA 014	Neolithic	J	44	1–20	rim	5	Rocker packed zigzags (stitches)	POM, ORA
SHA 032	Neolithic	L	59	15–30	rim	7	Rocker packed zigzags	POM, ORA
SHA 033	Neolithic	N	79	10–30	wall	4	Rocker packed zigzags	POM, ORA
SHA 034	Neolithic	I	60	(7A) 30	wall	7	Rocker spaced zigzags	POM, SEM, ORA
SHA 038	Neolithic	N	80	20–40	wall	8	WL incisions	POM, ORA
SHA 039	Neolithic	L	66	D	rim	6	Rocker packed curved zigzags (stitches)	POM, ORA
SHA 040	Neolithic	O	66	20–40	rim	6	Rocker packed zigzags (stitches)	POM, ORA
SHA 042	Neolithic	M	82	20–40	wall	5	Fish net	POM, ORA
SHA 043	Neolithic	N	66	1–20	rim	5	Rocker packed zigzags (stitches)	POM, SEM, ORA
SHA 044	Neolithic	M	83	70–100	wall	5	APS, DWL combined	POM, SEM, ORA
SHA 045	Neolithic	N	84–85	20–40	wall	6	Rocker "vees" and dots	POM, ORA
SHA 858	Neolithic	M	84	5–20	wall	6	Rocker Plain curved zigzags	POM, ORA
SHA 860	Neolithic	I	60		rim	5	Black topped	POM, ORA
SHA 866	Neolithic	J	60	30–50	wall	5	Rocker "vees" and dots	POM, ORA
SHA 877	Neolithic	N	88	5–20	wall	5	Rocker "vees" and dots	POM, ORA
SHA 887	Neolithic	M	80	20–40	wall	4	APS Double pronged	POM, ORA
SHA 891	Neolithic	I	60	10–40	rim	7	Rocker packed zigzags	POM, ORA
SHA 895	Neolithic	M	87	1–20	rim	7	Rocker "vees" and dots / decorated also inside	POM, ORA
SHA 897	Neolithic	N	82	5–20	rim	5	Undecorated	POM, SEM, ORA
SHA 909	Neolithic	L	83	khor	wall	5	Rocker "vees" and dots	POM, ORA
SHA 027	Late Neolithic	N	66	1–20	wall	7	Simple impression	POM, SEM, ORA
SHA 028	Late Neolithic	I	58	10–30	wall	5	Geometric	POM, ORA
SHA 029	Late Neolithic	N	66	1–20	wall	7	Geometric	POM, ORA
SHA 047	Late Neolithic	N	84	20–55	wall	5	Geometric	POM, ORA
SHA 049	Late Neolithic	I	59	20–40	bottom	9	Incisions	POM, ORA

As the ceramic material came from a museum collection and the area where the archaeological site was located has recently been affected by agricultural developments, it was not possible to access the local geological sediments in order to precisely determine the origin of the raw materials used to make the pottery. Thus, POM was employed to identify the likely geological provenance of clay raw materials and tempers and to provide information on the representative fabric types at the site, according to compositional and textural criteria.

Furthermore, scanning electron microscopy (SEM-EDS) was integrated with POM and applied to a selection of eight samples in order to validate the POM observations and identify specific technological choices with greater accuracy (Table 1). In particular, SEM-EDS analysis aimed to identify the presence (or absence) of phosphates in the pastes, which are potentially attributable to the intentional addition of herbivore dung to the clay or, possibly, to use contamination or post-depositional processes. A further important question concerned the identification of clay pellets, grog, and ferruginous aggregates in the pastes and the evidence of surface treatments, such as polishing, to improve the performance of the pots (e.g., impermeability).

Finally, the same 40 sherds selected for POM, together with a further 77 sherds (total 117) underwent organic residue analysis in order to understand the original function of the vessels and what commodities they may have processed. Combined with the reconstruction of dietary practices and food traditions, organic residue analysis from pottery lipids can provide ecological signatures and palaeoclimatic data on the ancient environment (see, e.g., [28–32]).

Full details on the methods used can be found in the Supplementary Material (S1 Text and S2 Table).

## Results

### Polarized optical microscopy (POM)

**Fig 9:**
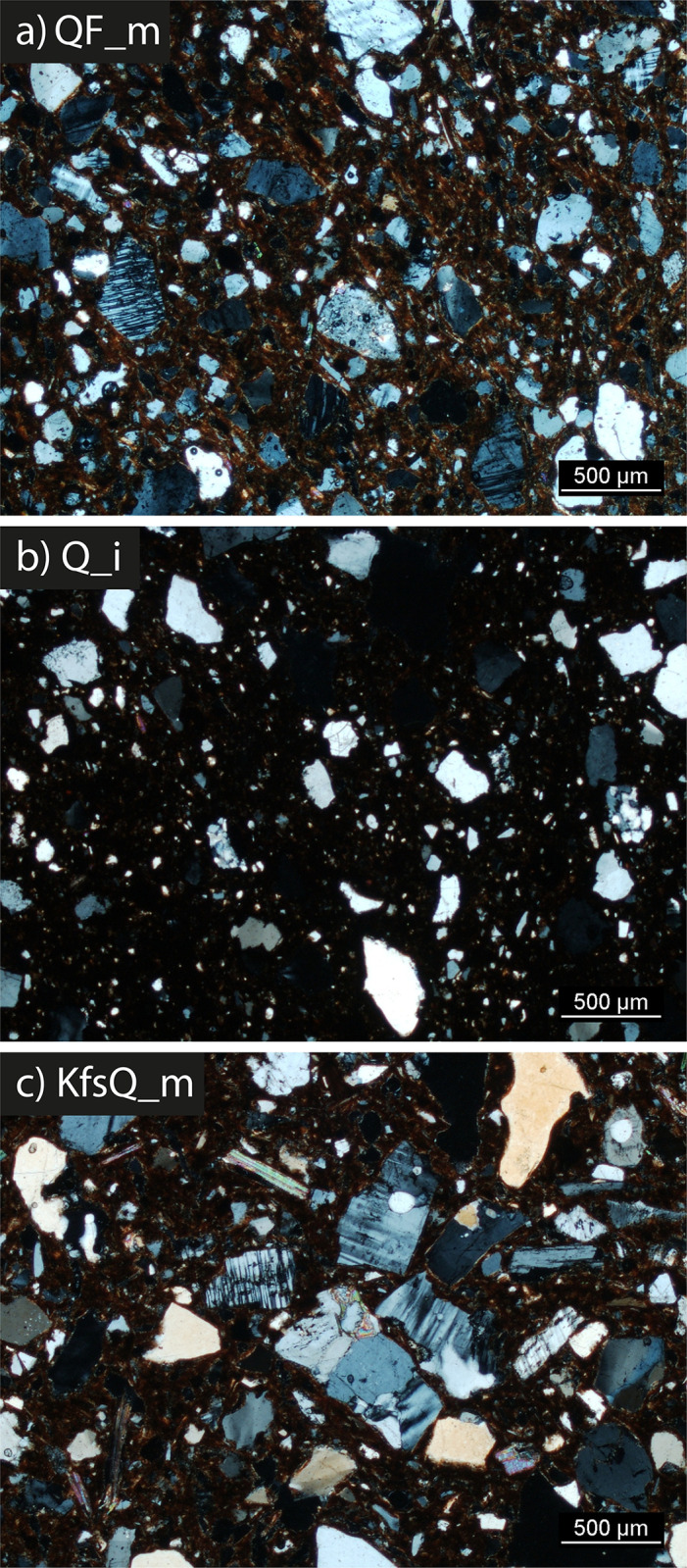
Photomicrographs of the main fabrics (POM): a) SHA039; b) SHA877; c) SHA058. Magnification 2.5x; polarization XP (Photos by G. Eramo).

The samples were petrographically differentiated according to three main fabrics: 1) Quartz and feldspars-rich (QF*); 2) Quartz-rich (Q*); and 3) K-feldspar and quartz-rich (KfsQ_m). Fabric QF* is the most frequent in the Early Khartoum sample and also characterizes the Neolithic and Late Neolithic ceramics. Conversely, the proportions are reversed in these later periods, with the majority having a quartz-rich (Q*) fabric and a minority containing alkali feldspar and granite rocks (see discussion). Table 2 and Fig 9 summarize the main petrographic features of the investigated samples.

**Table 2:** Petrographic (POM) outline of the pottery fabrics (i: Iron oxides-oxyhydroxides; k: Carbonates; m: Micaceous; o: Organic matter; cp: Clay pellets; Bir: Matrix birefringence; Str: Oxidation pattern; Txt: Texture; P1 and P2: Primary and secondary porosity; D mode: Prevalent grain size class; D max: Maximum grain size; H: Paste homogeneity; R: Grain roundness (0–4); Qm: Monocrystalline quartz, straight extinction; Qmu: Monocrystalline quartz, undulose extinction; Qp: Polycrystalline quartz; Ch: Chert; Pl: Plagioclase; Kfs: K-feldspars; Ms: Muscovite; Bt: Biotite; Hbl: Horneblend; Epi: Epidote; Zrn: Zircon; Rtl: Rutile; PRF: Plutonic rock fragments; SRF: Sedimentary rock fragments; VRF: Volcanic rock fragments; Ia: Ferruginous aggregates; Cal2: Secondary calcite; 0–3: Relative amounts or degree, when otherwise not specified; ?: Non detectable; ^#^: Altered).

					Matrix							Pores			Non plastic inclusions																							
																																						
GROUP	Sample	Period	Thickness	Fabric	i	k	m	o	cp	Bir	Str	Vol%	P1	P2	Vol%	Txt	D mode (μm)	D max (μm)	R	H	Qm	Qmu	Qp	Ch	Pl	Kfs	Ms	Bt	Hbl	Epi	Zrn	Rtl	PRF	SRF	VRF	Ia	Cal2	Temper
2	SHA014	Neo	5	Q_m	1	0	2	1	0	3	RO	15	2	2	25	B	32–63; 250–500	880	0–1	2	2	2	3	0	2	1	0	2	0	0	1	0	2	1	0	1	0	1
1B	SHA022	EK	9	KfsQ_m	1	0	2	0	0	3	O	10	2	1	25	B	63–125; 500–1000	1160	0–1	1	2	2	2	0	2	3*	1	2	0	1	1	1	1	0	0	2	0	0
1B	SHA023	EK	10	KfsQ_m	1	0	2	0	0	2	O	10	2	1	40	B	63–125; 500–1000	1100	0–1	2	2	2	2	0	1	3*	1	2	0	1	0	0	1	0	0	1	1	0
1D	SHA024	EK	7	QF_m	1	0	2	1	0	3	RO	15	2	1	40	S	250–500	1120	0–2	3	2	2	2	0	1	3	2	1	1	2	0	0	2	0	0	1	0	0
1D	SHA025	EK	8	KfsQ_m	1	0	2	1	0	2	RO	5	2	1	45	S	500–1000	1050	0–2	3	2	3	2	0	1	3*	2	1	0	1	0	0	2	1	0	1	0	0
1A	SHA026	EK	9	QF_m	1	0	2	2	0	2	ROE	10	2	2	30	S	250–500	1050	0–1	3	2	2	2	0	1	3	2	2	0	1	0	0	1	0	0	1	0	0
1	SHA027	Late Neo	7	QF_m	2	0	2	1	0	3	RO	15	2	2	40	S	250–500	620	0–1	3	2	2	2	0	1	2	2	3	1	1	0	0	1	0	0	2	1	0
2	SHA028	Late Neo	5	Q_i	2	0	1	2	1	2	R	15	3	1	20	B	32–63; 250–500	600	0–1	0	2	2	2	1	1	1	2	1	0	2	1	0	1	1	0	2	0	1
1	SHA029	Late Neo	7	QF_m	2	0	2	0	0	3	O	15	2	2	25	S	250–500	600	0–1	2	2	2	1	0	1	2	1	2	1	1	0	0	1	0	0	2	0	0
1	SHA032	Neo	7	QF_m	2	0	2	1	0	2	RO	10	1	2	35	S	63–125; 500–1000	1800	0–2	3	2	2	2	0	1	3	1	1	0	1	0	0	1	0	0	2	1	1
2	SHA033	Neo	4	Q_i	1	0	1	1	1	2	R	10	2	1	20	B	32–63; 250–500	720	0–1	1	2	2	3	1	1	1	1	0	1	2	1	0	2	1	0	2	0	1
2	SHA034	Neo	7	Q_i	2	0	1	1	1	2	R	15	3	1	20	B	32–63; 250–500	800	0–1	1	2	2	3	1	1	1	2	0	0	2	0	0	1	1	0	2	0	1
2	SHA038	Neo	8	Q_m	1	0	2	0	2	3	O	15	3	1	25	B	32–63; 250–500	2200	0–2	2	2	2	3	2	1	1	2	1	1	2	0	1	1	2	0	2	0	1
1	SHA039	Neo	6	QF_m	1	0	2	1	0	3	ROE	10	1	2	40	S	250–500	850	0–3	2	2	2	2	0	2	3	2	2	0	1	0	0	1	0	0	1	0	0
1	SHA040	Neo	6	QF_m	1	0	2	0	0	2	O	5	2	1	35	S	250–500	1050	0–1	3	2	2	2	0	2	3	1	1	0	1	0	0	1	0	0	1	0	0
2	SHA042	Neo	5	Q_m	1	0	2	1	1	2	R	15	3	2	25	B	32–63; 250–500	1000	0–1	2	3	2	2	2	1	1	1	0	0	1	0	0	1	2	0	2	0	1
1	SHA043	Neo	5	QF_m	1	0	2	1	1	3	RO	15	2	3	30	S	250–500	850	0–3	2	2	2	3	0	1	2	2	2	0	1	1	1	1	0	0	2	0	1
2	SHA044	Neo	5	Q_m	1	0	2	1	0	3	R	15	3	2	25	B	32–63; 250–500	600	0–1	2	3	2	2	1	1	1	1	0	0	2	0	0	2	1	0	1	0	1
1	SHA045	Neo	6	QF_m	1	0	2	1	0	3	O	15	3	1	40	S	250–500	840	0–2	3	2	2	2	0	3*	3	2	2	1	2	0	0	2	0	0	2	0	0
2	SHA047	Late Neo	5	Q_i	2	0	1	1	1	2	RO	15	2	2	35	B	32–63; 250–500	480	0–1	2	3	2	2	0	1	1	1	1	0	2	1	1	2	1	1	2	0	1
2	SHA049	Late Neo	9	Q_i	1	0	1	1	1	3	ROI	15	2	2	35	B	32–63; 250–500	880	0–1	2	3	2	2	1	1	1	1	1	0	2	1	0	2	1	0	2	0	1
3	SHA050	EK	9	QF#_m	1	0	2	1	0	2	RO	15	2	3	35	S	250–500	640	0–2	3	3	2	2	1	2*	3*	1	2	1	1	1	1	2	1	0	1	0	0
1B	SHA051	EK	10	QF_m	2	0	2	0	1	2	RO	15	2	2	30	S	63–125; 500–1000	1150	0–1	2	2	2	1	0	1	3	1	2	1	1	0	0	1	0	0	2	1	1
1B	SHA052	EK	8	QF_i	2	0	1	0	1	2	O	10	2	1	40	S	250–500	1110	0–1	2	2	2	1	0	1	3	1	2	1	1	0	0	1	0	0	2	0	0
1C	SHA053	EK	12	KfsQ_m	2	0	2	1	1	2	R	15	3	1	40	B	63–125; 500–1000	1720	0–1	2	2	2	1	0	1	3*	1	2	1	1	1	0	1	0	0	2	0	0
1C	SHA054	EK	6	KfsQ_m	1	0	2	0	1	2	RO	20	3	1	35	B	63–125; 500–1000	1590	0–1	2	2	2	1	0	1	3*	1	2	0	1	0	0	1	0	0	2	0	0
1A	SHA055	EK	8	QF_m	1	0	2	1	0	3	ROE	10	2	1	35	S	250–500	800	0–3	3	2	2	2	0	2	3*	1	1	0	1	0	0	1	0	0	2	0	0
2	SHA056	EK	7	Q_i	2	0	1	2	1	2	R	20	2	3	30	B	32–63; 250–500	720	0–1	2	3	2	2	1	1	1	1	0	1	1	0	0	1	1	0	2	0	1
1D	SHA057	EK	9	QF_m	2	0	2	2	0	2	ROE	20	3	2	40	S	250–500	1010	0–2	3	2	2	2	0	2	3	2	1	0	2	0	0	1	0	0	2	0	0
1D	SHA058	EK	7	KfsQ_m	1	0	2	1	0	2	O	20	3	2	40	S	500–1000	1060	0–2	3	2	2	2	0	2	3	2	1	0	1	1	0	1	0	0	1	0	0
2	SHA059	EK	7	Q_i	1	0	1	0	0	2	O	15	3	2	30	B	32–63; 250–500	1600	0–2	2	3	2	2	1	2	1	1	0	1	2	0	0	2	1	0	2	0	1
2	SHA858	Neo	6	Q_i	2	0	1	0	2	2	O	10	2	1	25	B	32–63; 250–500	720	0–2	2	3	2	2	1	1	1	0	0	1	1	0	0	1	1	0	2	1	1
1	SHA860	Neo	5	QF_i	2	0	1	1	1	2	ROE	10	2	1	35	B	32–63; 250–500	920	0–2	2	2	2	1	0	1	2	1	2	0	1	0	0	1	0	0	2	0	1
2	SHA866	Neo	5	Q_i	2	0	1	1	1	2	RO	10	2	1	35	B	32–63; 250–500	670	0–1	2	3	2	2	1	1	1	1	1	0	1	1	0	2	1	1	2	0	1
2	SHA877	Neo	5	Q_i	2	0	1	1	1	2	ROI	10	2	1	35	B	32–63; 250–500	920	0–2	1	2	2	1	2	1	2*	1	0	0	1	0	0	1	0	0	2	0	1
1	SHA887	Neo	4	QF_i	2	0	1	1	1	2	R	10	2	1	30	B	32–63; 250–500	920	0–2	2	3	2	2	2	1	1	1	0	0	1	1	0	1	2	0	2	0	1
2	SHA891	Neo	7	Q_i	2	0	1	1	1	2	RO	10	2	1	35	B	32–63; 250–500	920	0–1	2	3	2	2	1	1	1	1	0	0	1	0	0	1	1	0	2	0	1
1	SHA895	Neo	7	QF_m	2	0	2	1	0	2	RO	10	2	1	35	S	250–500	790	0–2	2	2	2	2	0	1	3*	1	0	0	1	0	0	1	0	0	2	0	1
1	SHA897	Neo	5	QF#_m	2	0	2	1	0	3	O	10	2	1	35	S	250–500	700	0–2	3	2	2	2	0	2*	3*	1	2	1	2	0	0	2	0	0	2	0	0
2	SHA909	Neo	5	Q_i	2	0	1	0	1	2	O	10	2	1	30	B	32–63; 250–500	1200	0–1	2	3	2	2	2	1	1	1	0	0	1	1	0	1	2	0	2	0	1

#### Fabric QF*

This fabric includes the largest number of samples, totalling 18 (SHA024, SHA026, SHA27, SHA029, SHA032, SHA039, SHA040, SHA043, SHA045, SHA050, SHA051, SHA052, SHA055, SHA057, SHA860, SHA887, SHA895, and SHA897).

NPIs are characterized by monocrystalline quartz with straight and undulose extinction and polycrystalline quartz in similar proportions. Among feldspars, microcline is prevalent on plagioclase and, in a few cases, feldspars show intense alteration (SHA050 and SHA897). Biotite and muscovite are diffused in both the NPIs and the matrix. Epidote occurs in traces in all the samples, whereas hornblende was identified only in seven thin sections. A few crystals of rutile and zircon were observed. Traces of granitic rock fragments are present in all the potsherds, as well as ferruginous aggregates (Fig 9A). A co-occurrence of chert grains and sedimentary rock fragments was identified in SHA050 and SHA887. As a whole, NPIs were estimated in 25–40% vol. The maximum sizes of the grains range between 600 μm and 1800 μm, and the grain-size distribution is prevalently unimodal and seriate. The homogeneity (H) of NPIs distribution is quite high. Grain roundness (R) is mostly medium (dominant range between 0–2), even though some samples have a lower roundness of NPIs (range 0–1) and only a minority of them show a greater roundness (range 0–3). In general, most NPls, and especially the alkali feldspars are characterized by angular or sub-angular shapes, while quartz has more often rounded margins.

The matrix is relatively rich in micas (samples SHA024, SHA026, SHA027, SHA029, SHA032, SHA039, SHA040, SHA043, SHA045, SHA050, SHA051, SHA055, SHA057, SHA895, and SHA897 labeled as QF_m variants) and iron oxides (samples SHA052, SHA860, and SHA887 labeled as QF_i variants). Clay pellets (cp) are rarely attested. Partial oxidation patterns (RO, ROE) are more frequent than a complete oxidation (O) and reveal variable amounts of carbonaceous matter dispersed in the matrix. A medium to high birefringence of the matrix is the rule. Most of the samples show a pore fraction of 10–15% vol., with a prevalence of primary over secondary porosity.

#### Fabric Q*

This fabric accounts for 16 samples (SHA014, SHA028, SHA033, SHA034, SHA038, SHA042, SHA044, SHA047, SHA049, SHA056, SHA059, SHA858, SHA866, SHA877, SHA891, and SHA909), all characterized by a bimodal grain-size distribution, together with varied maximum grain sizes (480–2200 μm). The NPI content is relatively lower than in the other two fabrics (20–35% vol.). A lower homogeneity of NPIs distribution and grain roundness (dominant range between 0–1) was observed. Polycrystalline or monocrystalline quartz is prevalent, with straight and undulose extinction. Feldspars are less abundant than in the other two fabrics. Variable amounts of epidote occur in all the samples, whereas few crystals of hornblende, rutile, and zircon were identified. Muscovite exceeds biotite in the NPIs. Traces of granite and sandstone fragments occur in all the samples. Ferruginous aggregates are quite frequent (Fig 9B).

Iron oxi-hydroxides (samples SHA028, SHA033, SHA034, SHA047, SHA049, SHA056, SHA059, SHA858, SHA866, SHA877, SHA891, and SHA909 labeled as Q_i variants) and small crystals of micas (samples SHA014, SHA038, SHA042, and SHA044 labeled as Q_m variants) are dispersed in the matrix. Few clay pellets were identified in almost every thin section. Some carbonaceous matter is dispersed in the matrix. The number of non-oxidized (R) samples equals that of partial oxidation patterns (RO, ROI) and exceeds that of complete oxidation (O). Where detectable, birefringence shows a prevalent medium degree. Porosity accounts for 10–20% vol., and primary pores are more common than secondary ones.

### Fabric KfsQ_m

This fabric consists of 6 samples (SHA022, SHA023, SHA025, SHA053, SHA054, and SHA058). Estimation of NPIs was between 25 and 45% vol. The grain-size distribution of the NPIs is prevalently bimodal, with higher homogeneity of distribution in unimodal seriate samples (SHA025 and SHA058). The maximum sizes of the grains range between 1050 μm and 1720 μm. Roundness is low. Altered K-feldspars are often the prevalent component of this fabric. As in fabric QF*, monocrystalline quartz with straight and undulose extinction and polycrystalline quartz occur in similar proportions. Biotite is more abundant than muscovite, and both are also present in the matrix. Traces of epidote and granitic rock fragments are present in all the samples. Rutile, zircon, and sandstone grains are rare (Fig 9C).

A micaceous matrix with few clay pellets was observed. Traces of carbonaceous matter occur in the matrix. Complete oxidation (O) of the matrix and medium birefringence are dominant. Most of the samples show a pore fraction of 5–20% vol., with a prevalence of primary over secondary porosity.

### Scanning electron microscopy and energy dispersive spectroscopy (SEM-EDS)

**Fig 10:**
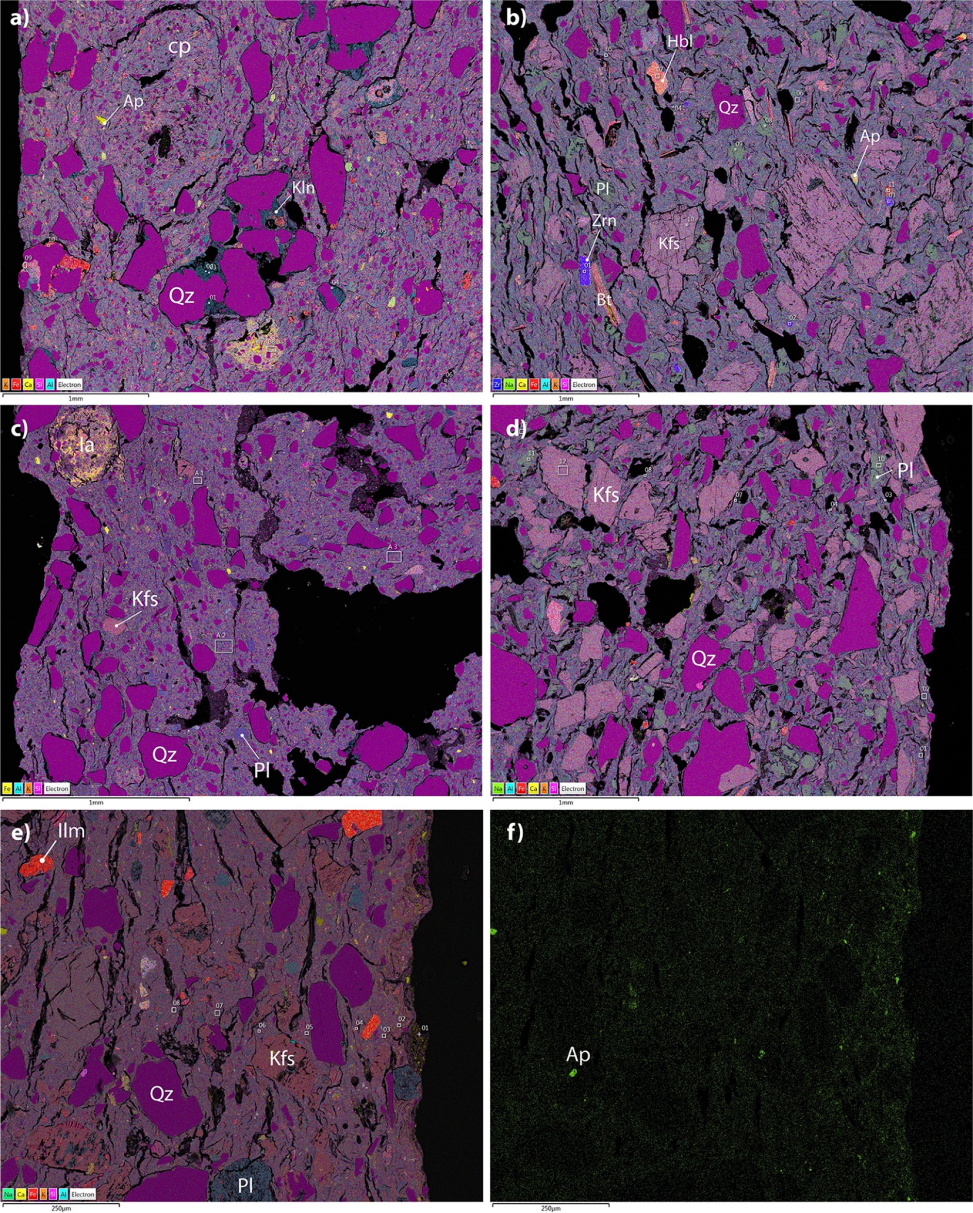
Layered elemental maps (SEM-EDS) of representative samples of the main fabrics: a) SHA034 (Q_i); b) SHA053 (KfsQ_m); c) SHA044 (Q_i); d) SHA025 (KfsQ_m); e) SHA050 (QF_m) and the single map of P (f) of the same frame. Mineral abbreviations after [68] (Photos by G. Eramo).

A total of 9 samples (SHA 025, SHA 027, SHA 034, SHA 043, SHA 044, SHA 050, SHA 053, SHA 056, SHA 897) representing the three fabrics were analyzed with SEM-EDS (Table 3). EDS microanalysis revealed an albitic composition of plagioclases. Very small particles of rutile and epidote are dispersed in the matrix of all the samples. Other heavy minerals of plutonic origin are apatite (SHA025, SHA027, and SHA056) and ilmenite (SHA027, SHA053, and SHA897). EDS analysis confirms the Ca-poor clay matrix (Fig 10A–10E). The presence of phosphates associated with possible dung addition was excluded, as was the presence of grog.

**Table 3:** Mean values and standard deviations (*italics*) of EDS analyses of the clay-rich portion of the matrix. In parenthesis: number of raster analyses (ca. 20x20 μm^2) per thin section. All data are normalized. Full data in S3 Table.

Sample	Na_2_O	MgO	Al_2_O_3_	SiO_2_	P_2_O_5_	K_2_O	CaO	TiO_2_	Fe_2_O_3_
SHA025 (*n* = 9)	0.3	2.0	26.1	57.8	0.4	3.1	3.0	0.5	6.8
	*0*.*03*	*0*.*23*	*1*.*87*	*0*.*93*	*0*.*31*	*0*.*67*	*0*.*45*	*0*.*17*	*0*.*86*
SHA027 (*n* = 7)	0.3	1.8	26.3	55.1	0.1	3.3	3.1	1.0	9.1
	*0*.*05*	*0*.*03*	*1*.*06*	*0*.*46*	*0*.*23*	*0*.*32*	*0*.*33*	*0*.*15*	*0*.*60*
SHA034 (*n* = 5)	0.4	1.6	27.0	56.4	0.3	1.9	2.2	1.0	9.2
	*0*.*26*	*0*.*23*	*4*.*25*	*1*.*03*	*0*.*26*	*0*.*28*	*1*.*03*	*0*.*67*	*2*.*95*
SHA043 (*n* = 10)	0.7	1.6	25.6	57.1	0.3	2.4	3.1	0.9	8.3
	*0*.*05*	*0*.*72*	*0*.*79*	*2*.*25*	*0*.*15*	*0*.*97*	*1*.*28*	*0*.*42*	*3*.*36*
SHA044 (*n* = 8)	1.3	1.8	21.5	58.3	0.2	2.2	2.7	1.4	10.6
	*0*.*40*	*0*.*18*	*0*.*61*	*1*.*14*	*0*.*04*	*0*.*21*	*0*.*13*	*0*.*07*	*0*.*30*
SHA050 (*n* = 12)	0.8	1.2	23.6	53.6	1.6	4.1	4.6	0.5	10.1
	*0*.*20*	*0*.*30*	*2*.*17*	*3*.*50*	*0*.*61*	*2*.*96*	*4*.*05*	*0*.*22*	*2*.*14*
SHA053 (*n* = 6)	0.6	1.4	26.1	54.8	0.6	3.5	3.3	0.9	8.8
	*0*.*57*	*0*.*13*	*0*.*50*	*1*.*36*	*0*.*11*	*0*.*51*	*0*.*50*	*0*.*15*	*1*.*04*
SHA056 (*n* = 7)	0.5	1.5	27.7	56.1	0.4	1.8	2.0	1.1	9.1
	*0*.*23*	*0*.*23*	*9*.*68*	*3*.*43*	*0*.*24*	*0*.*35*	*1*.*20*	*0*.*64*	*3*.*58*
SHA897 (*n* = 7)	0.4	1.7	23.5	53.9	0.5	2.9	4.7	0.9	11.5
	*0*.*17*	*0*.*03*	*2*.*59*	*0*.*58*	*0*.*27*	*0*.*73*	*0*.*76*	*0*.*01*	*1*.*11*

**Fig 11:**
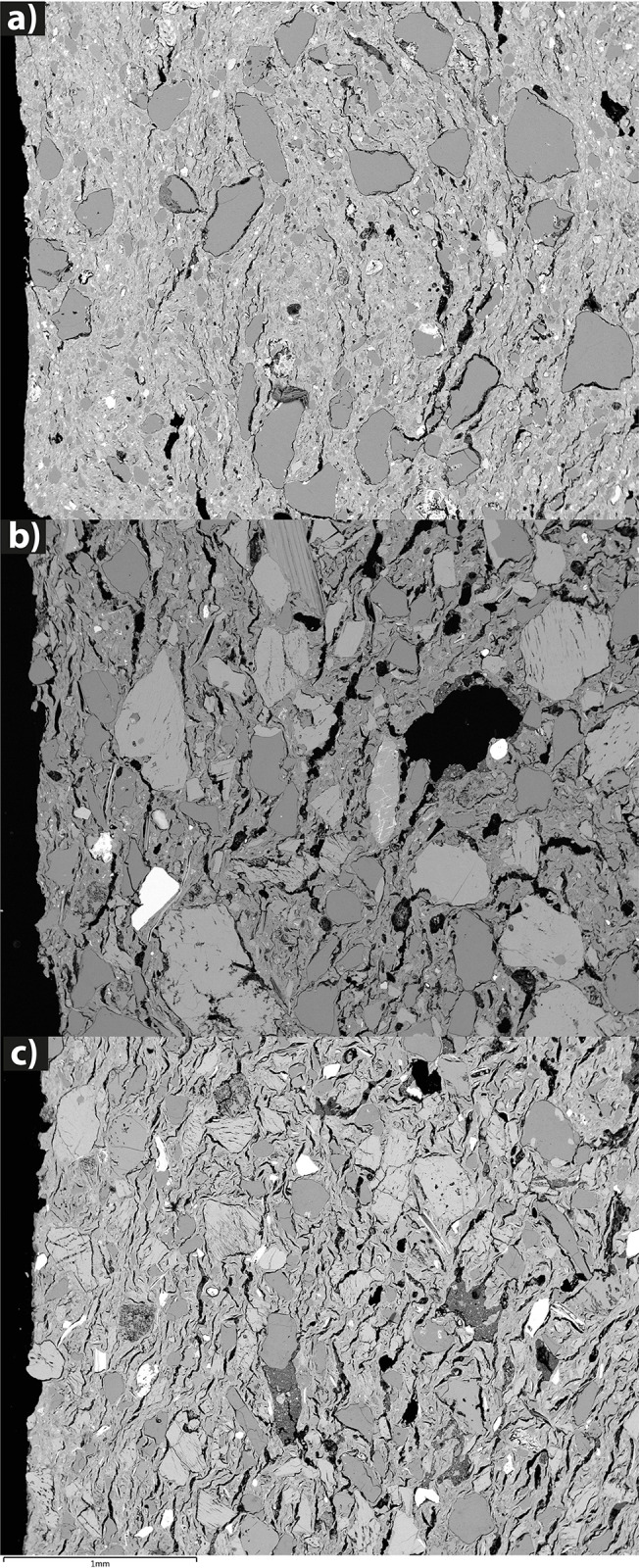
SEM BSE images showing the microstructures of three representative samples of the (a) fabric Q_i (SHA034); (b) fabric KfsQ_m (SHA025); (c) fabric QF_m (SHA897).

The matrix of fabric Q* contains silt-size feldspars, whereas quartz grains in the NPIs have a medium sand matrix (Fig 10A and 10C). Diffused Ca, Mg, and K in the matrix account for smectite-illite clay, which is different from the kaolinitic clay present in some quartz aggregates. Frequently, clay pellets have no quartz sand, which is a strong clue for tempering (Fig 10A). An aggregate enriched in Ca, Mn, and P was identified in SHA034 (Neolithic). Faint P contamination was detected on the surface of SHA056 (Early Khartoum). BSE images reveal some compactness of the ceramic body near the surface (about 1mm). A relative enrichment in the micaceous component of the matrix of SHA044 (Q_m), as observed with POM, is confirmed by EDS analyses (Table 3). In this quartz-rich fabric, the secondary pores (P2) are less present than in the other two fabrics (Fig 11A).

Fabric KfsQ_m (Early Khartoum) has abundant coarse-sized alkali feldspars and biotite with a few smaller plagioclases (Fig 10B and 10D). Rare hornblende crystals were detected (Fig 10B). The matrix consists of prevalent smectite-illite clay, like Fabric Q*, but is more clayey. NPIs show a continuous range in grain size (seriate texture), and the mineralogical content of the NPIs is consistent with that of the matrix. Secondary lenticular pores (P2) are spread throughout the body of the sample.

The analyzed samples of Fabric QF* share a seriate texture with variable amounts of NPIs. In this case, quartz is prevalent over feldspars (Fig 10E). No anomalous concentrations of P were detected in the paste, except for SHA050, where Ca-phosphates contamination on the inner (lower) and outer surface (higher) occurs (Fig 10F), and a mean P_2_O_5_ concentration of 1.64% wt. was calculated (Table 3). Minor amounts of ilmenite grains are present in all QF* thin sections (Fig 10E). As in KfsQ_m samples, biotite and muscovite are diffused in the NPIs, and the matrix is based on smectite-illite clay. As for KfsQ_m, secondary pores are quite frequent and widespread. However, a more compact body next to the surface than inward was observed in SHA897 (Neolithic) (Fig 11C).

### Organic residue analysis (ORA)

**Fig 12:**
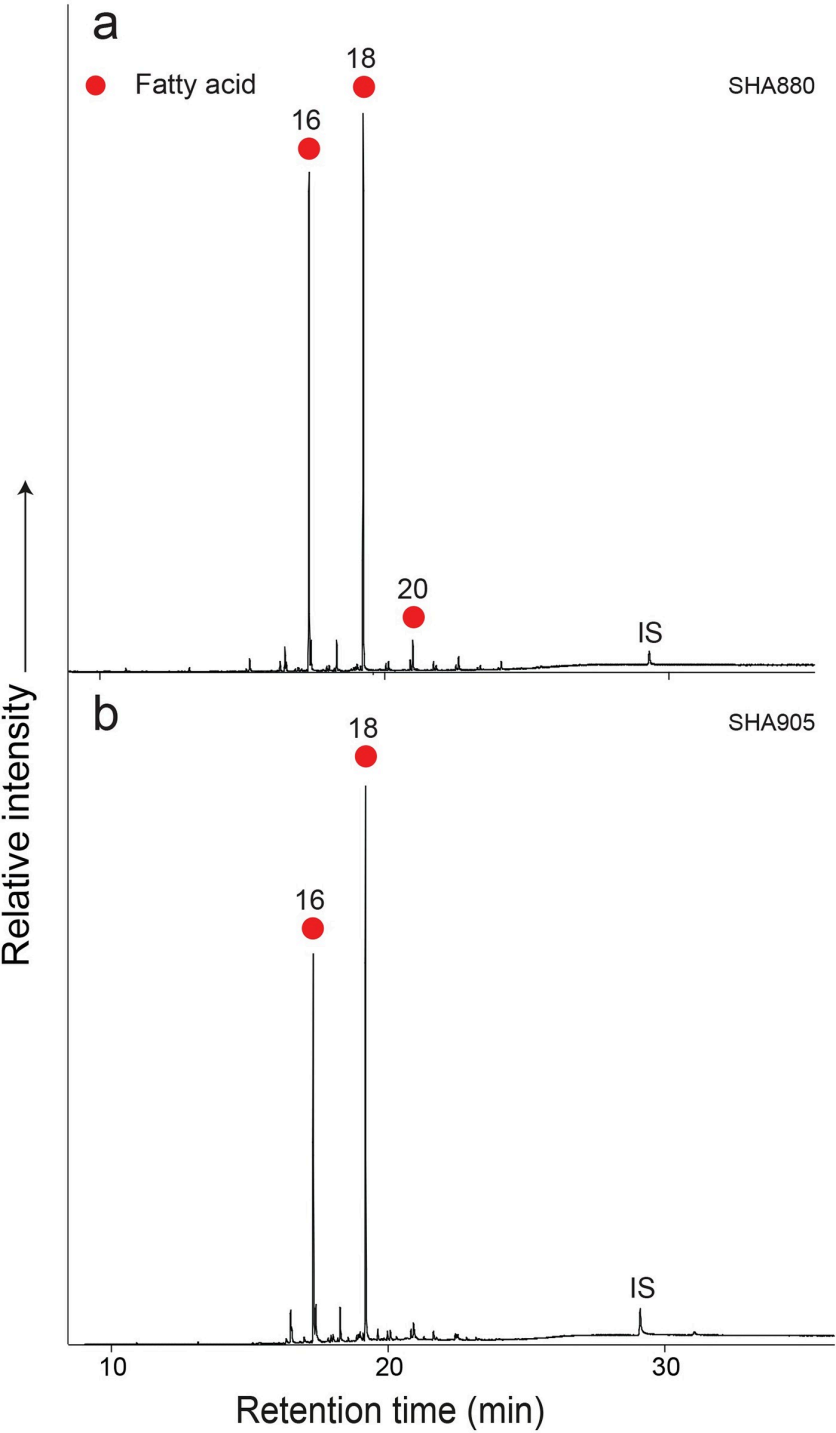
Partial gas chromatograms of trimethylsilylated FAMEs from the Esh-Shaheinab pottery extracts of **a**. SHA880 and **b**. SHA905, red circles, n-alkanoic acids (fatty acids, FA), IS, internal standard, C_34_ n-tetratriacontane. Numbers denote carbon chain length.

**Fig 13:**
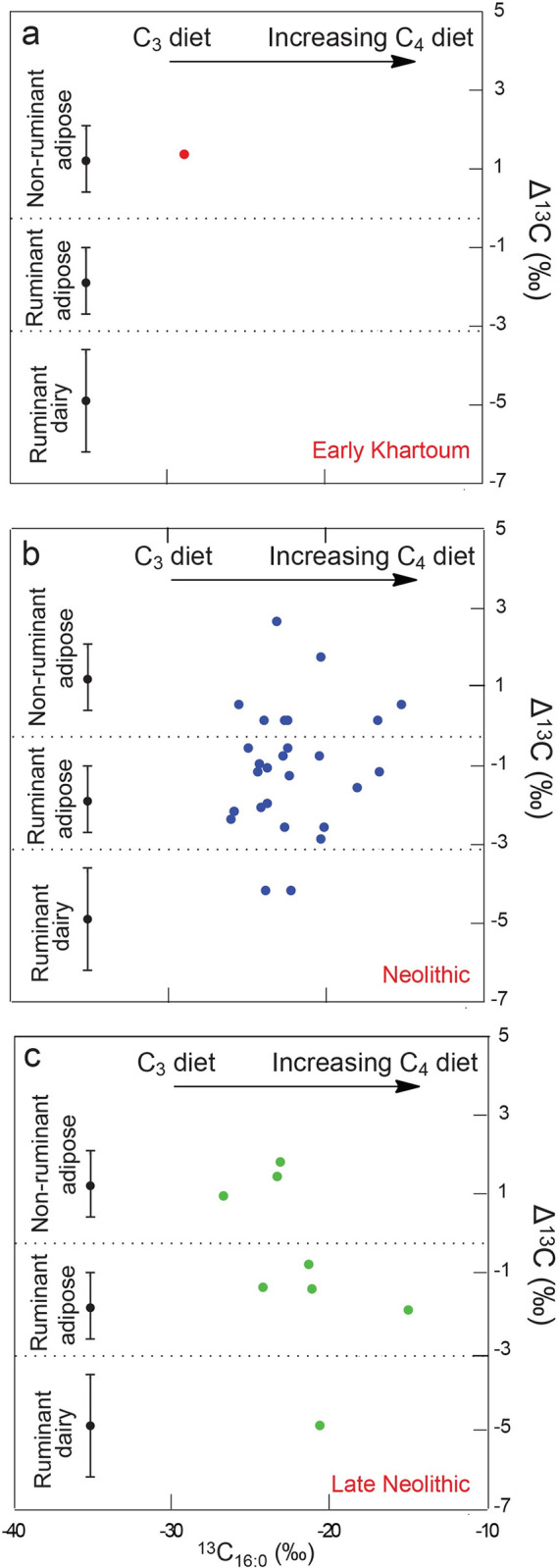
Graphs showing: Δ^13^C (δ^13^C_18:0 –_δ^13^C_16:0_) values from a. Early Khartoum, b. Neolithic and c. Late Neolithic vessels from Esh-Shaheinab. Ranges shown here represent the mean ± 1 s.d. of the Δ^13^C values for a global database comprising modern reference animal fats from the UK, Africa and elsewhere [28, 69].

A total of 117 sherds were analyzed: 20 from the Early Khartoum period, 86 from the Neolithic, and the remaining 11 from the Late Neolithic. Of these, 36 sherds (31% recovery overall) contained sufficient concentrations (>5μg g^-1^) of lipids that can be reliably interpreted [33], although lipid recovery varied across the periods, at 5% in the Early Khartoum (*n* = 1), 31% in the Neolithic (*n* = 27) and 73% in the Late Neolithic (*n* = 8). The mean lipid concentration from the sherds (Table 4) was 0.26 μg g^-1^, with a maximum lipid concentration of 0.92 μg g^-1^ (SHA880). The lipid profiles were dominated by the free fatty acids, palmitic (C_16_) and stearic (C_18_), typical of a degraded animal fat (Fig 12) (e.g., [34, 35]). GC-C-IRMS analyses were carried out on the 36 lipid-yielding samples (Table 4 and Fig 13) to determine the δ^13^C values of the major fatty acids, C_16:0_ and C_18:0_, and ascertain the source of the lipids extracted. The δ^13^C values of the C_16:0_ and C_18:0_ fatty acids reflect their biosynthetic and dietary origin, allowing non-ruminant and ruminant adipose and ruminant dairy products to be distinguished [28, 36]. Lipid residue results for the one lipid-yielding potsherd from the Early Khartoum period plots in the non-ruminant adipose region with a Δ^13^C value of 1.3 ‰ (Table 4 and Fig 13A).

**Table 4:** Laboratory number, square y axis, square x axis, layer, lipid concentration (μg g^-1^), δ^13^C and Δ^13^C values, attributions of pottery lipid residues, period and decoration.

Laboratory Number	Square y axis	Square x axis	Layer	Lipid concentration (μg g^-1^)	δ^13^C_16:0_	δ^13^C_18:0_	Δ^13^C	Attribution	Period	Decoration
SHA023	L	66	D	64,5	-28,8	-27,5	1,3	Non-ruminant adipose	Early Khartoum	Dotted wavy line
SHA004	J	60	30–50	314,6	-22,4	-22,4	0,1	Mixed ruminant/non-ruminant adipose	Neolithic	
SHA005	N	84–85	20–55	142,2	-22,4	-23,1	-0,6	Mixed ruminant/non-ruminant adipose	Neolithic	
SHA032	L	59	15–30	499,9	-22,2	-26,4	-4,2	Dairy fat	Neolithic	Rocker, packed zigzags
SHA033	N	79	10–30	420,3	-20,4	-21,2	-0,8	Mixed ruminant/non-ruminant adipose	Neolithic	Rocker, packed zigzags
SHA034	I	60	7(A) 30	100,0	-26,0	-28,4	-2,4	Ruminant adipose	Neolithic	Rocker, spaced zigzags
SHA038	N	80	20–40	86,4	-25,5	-24,9	0,5	Non-ruminant adipose	Neolithic	Wavy line
SHA039	L	66	D	417,9	-25,8	-28,1	-2,2	Ruminant adipose	Neolithic	Rocker, packed zigzags
SHA040	O	66	20–40	363,0	-24,1	-26,2	-2,1	Ruminant adipose	Neolithic	Rocker, packed zigzags
SHA042	M	82	20–40	103,0	-23,1	-20,5	2,6	Non-ruminant adipose	Neolithic	Fishnet
SHA043	N	66	1–20	155,4	-22,6	-25,2	-2,6	Ruminant adipose	Neolithic	Packed zigzags
SHA044	M	83	70–100	775,9	-22,6	-22,6	0,1	Mixed ruminant/non-ruminant adipose	Neolithic	APS, Dotted wavy line
SHA045	N	84–85	20–40	280,7	-16,7	-16,6	0,1	Mixed ruminant/non-ruminant adipose	Neolithic	Rocker, vees+dots
SHA858	M	84	5–20	270,1	-15,2	-14,7	0,5	Non-ruminant adipose	Neolithic	Rocker, plain zigzags
SHA860	I	60	-	279,1	-20,3	-23,2	-2,9	Mixed dairy/ruminant adipose	Neolithic	Black topped
SHA866	J	60	30–50	212,6	-20,1	-22,7	-2,6	Ruminant adipose	Neolithic	Rocker, vees+dots
SHA877	N	88	5–20	85,1	-24,9	-25,5	-0,6	Mixed ruminant/non-ruminant adipose	Neolithic	Rocker, vees+dots
SHA878	N	81	40–55	76,4	-23,7	-24,7	-1,1	Ruminant adipose	Neolithic	Undecorated
SHA880	M	66	60–80	924,4	-23,8	-28,0	-4,2	Dairy fat	Neolithic	
SHA887	M	80	20–40	54,2	-20,3	-18,6	1,7	Non-ruminant adipose	Neolithic	APS, dots
SHA888	K	60	60–80	422,3	-18,0	-19,6	-1,6	Ruminant adipose	Neolithic	APS, dotted wavy line
SHA891	I	60	10–40	358,2	-23,9	-23,8	0,1	Mixed ruminant/non-ruminant adipose	Neolithic	Rocker, packed zigzags
SHA895	M	87	1–20	198,3	-24,3	-25,6	-1,2	Ruminant adipose	Neolithic	Rocker, vees+dots
SHA897	N	82	5–20	37,3	-22,3	-23,7	-1,3	Ruminant adipose	Neolithic	Undecorated
SHA905	M	66	40–60	112,2	-16,6	-17,8	-1,2	Ruminant adipose	Neolithic	APS, dashes
SHA907	N	80	20–40	295,4	-23,7	-25,8	-2,0	Ruminant adipose	Neolithic	APS, dots, panelled
SHA903	M	79	S	184,6	-22,7	-23,6	-0,8	Mixed ruminant/non-ruminant adipose	Neolithic	APS, dashes
SHA909	L	83	khor	81,0	-24,2	-25,2	-1,0	Ruminant adipose	Neolithic	Rocker, vees+dots
SHA008	J	59	20-40D	128,8	-21,1	-22,4	-1,3	Ruminant adipose	Late Neolithic	
SHA009	M	84	5–20	105,5	-24,2	-25,5	-1,3	Ruminant adipose	Late Neolithic	
SHA010	I	60	40–60	254,8	-21,3	-22,0	-0,7	Ruminant adipose	Late Neolithic	
SHA027	N	66	1–20	174,8	-23,3	-21,8	1,5	Non-ruminant adipose	Late Neolithic	Simple impression
SHA029	N	66	1–20	175,7	-26,7	-25,7	1,0	Non-ruminant adipose	Late Neolithic	Geometric
SHA046	I	58	(3)	280,5	-15,0	-16,9	-1,9	Ruminant adipose	Late Neolithic	Incision, stylus, panelled
SHA047	N	84	20–55	152,6	-20,6	-25,4	-4,8	Dairy fat	Late Neolithic	Geometric
SHA048	I	60	D	701,0	-23,1	-21,2	1,9	Non-ruminant adipose	Late Neolithic	Ripple
										

The majority of Neolithic vessels were used to process solely ruminant carcass products (*n* = 12, 44%) with a further 30% (*n* = 8) being used in cooking mixtures of ruminant and non-ruminant products, whether from domesticated or wild ruminant fauna. Four Neolithic vessels (15%) were used in processing solely non-ruminant products. In the Late Neolithic, a greater number of vessels (*n* = 4, 50%) were used to process ruminant carcass products, with 38% (*n* = 3) being used for cooking non-ruminant products. However, it must be noted that this is a smaller dataset. The δ^13^C_16:0_ values of the fatty acids from the Neolithic period (*n* = 27) range from -26.0 to -15.2 ‰ with the δ^13^C_18:0_ values ranging from -28.4 to -14.7 ‰ (Table 4), producing a broad range (-10.8 ‰ and -13.7 ‰ for the δ^13^C_16:0_ and δ^13^C_18:0_, respectively) (Table 4 and Fig 13B). Similarly, the δ^13^C_16:0_ values of the fatty acids from the Late Neolithic period (*n* = 8) range from -26.7 to -15.0 ‰ and the δ^13^C_18:0_ values range from -25.7 to -16.9 ‰ (Table 4). This broad range (-11.7 ‰ and -8.8 ‰ for the δ^13^C_16:0_ and δ^13^C_18:0_, respectively) across the Neolithic and Late Neolithic suggests the animals producing these fats consumed an extremely wide range of plant diets either composed mainly of C_3_ plants, varying combinations of C_3_ and C_4_ plants to a diet comprising primarily C_4_ plants (Fig 13B and 13C).

Twenty-one of the lipid-yielding samples were analyzed by gas chromatography-mass spectrometry (GC-MS) in selected ion monitoring (SIM) mode to check for the presence of freshwater biomarkers, such as *ω*-(*o*-alkylphenyl) alkanoic acids (APAAs) and vicinal dihydroxy acid (DHYAs), which denote the processing of shellfish/crustaceans, fish, waterfowl and aquatic mammals (see [37]). No aquatic biomarkers were detectable in the analyzed potsherds, and although some aquatic input to the vessels cannot be discounted, these results hint that fish were not boiled in pots at Esh-Shaheinab.

Also noteworthy is the absence of lipid evidence for the processing of plants, such as wild grasses, leafy plants, and sedges, found in other Sudanese Mesolithic and Neolithic vessels at sites such as Khor Shambat and al-Khiday [31, 32].

## Discussion

The results of the petrographic analysis by POM and SEM-EDS are discussed below according to the various stages of the *chaîne opératoire*, starting with the procurement of the clay raw materials and tempers (provenance), through the following stages of the manufacturing sequence, including processing, forming, and firing. The results of the lipid analysis are discussed and interpreted with regard to the use and function of the ceramic vessels.

### Provenance

The petrographic analysis highlights different degrees of clay processing in the three fabrics, with the presence of different amounts of granitic rock fragments in a Ca-poor clay matrix as their common denominator. This shows that a parent crystalline rock existed in the sedimentary basin where these raw materials were acquired. The petrofacies signature of fabrics KfsQ_m and QF* indicates that sediments were close to the parent granitic rock. In particular, fabric KfsQ_m has a coarser texture than QF*, with a lower degree of roundness of the NPIs and a wider range of grain sizes. SHA014 shows intermediate characteristics between fabric Q* and fabric KfsQ_m. Monocrystalline and polycrystalline quartz dominates in this sample but there is also considerable biotite, some K-feldspar, and plutonic rock fragments (granite). The three fabrics contain rare volcanic lithic fragments and, in general, minerals related to a volcanic component (i.e., pyroxenes) as NPIs (Table 2). Conversely, the impoverishment of micas and feldspars and the presence of a sedimentary component (chert and sandstones) denote a different supply area for the samples attributed to the Q* fabric. Organic plant inclusions are scanty in this fabric, except for SHA909 (fabric Q_i), which can be considered as an endmember of this fabric.

The grain sizes of the samples are consistent with those of the fluvial deposits (levees and sand bars) of the Nile between Khartoum and Atbara, where fine to medium sands are attested and may locally reach a few millimeters in diameter [38]. The petrofacies in this section of the Nile result from sedimentary inputs from the White Nile and the Blue Nile that converge in the Khartoum area. The main sediment supply to the Nile comes from the Blue Nile, although the White Nile has the largest catchment area [39]. This also reflects a qualitative as well as quantitative difference in grain size and composition. The suspended load of the Blue Nile is responsible for the finer fraction of the fluvial sediments, dominated by a volcaniclastic component derived from the Ethiopian basaltic plateau, whereas bedload sand is largely derived from the gneissic Precambrian basement. The prevalence of smectite in the clay minerals accounts for the arid tropical climate that characterizes the Blue Nile and Atbara basins [38, 40].

The sediment contribution of the White Nile is marked by quartz and feldspar-rich sands with abundant amphibole and epidote that originate from crystalline basements in western Ethiopia drained by the Baro, Gila, and Akobo Rivers. These sediments change composition repeatedly in the East African Rift lakes and in the Sudd and Machar marshes, where sediment trapping strongly affects the amount and composition of the White Nile [39]. Downstream of the Sudd marshes, the coarser fraction of the sediments is dominated by monocrystalline quartz with little feldspar and reworked calcrete (caliche) grains. Only epidote and amphibole are still relatively frequent among heavy minerals. Downstream of Khartoum, some plagioclase, volcanic rock fragments, and clinopyroxene recycled from the Gezira Formation become part of the White Nile. Kaolinite represents the mineralogical signature of the humid equatorial catchment areas on the clay fraction, although the alkaline environment of the swampy lowlands in South Sudan makes smectite the prevalent clay mineral, with subordinate kaolinite, chlorite, and illite reducing differences in the composition of the fine portion of the Blue Nile [38, 41].

The petrographic and mineralogical content of the three pottery fabrics partially mirrors the alluvial Nile deposits mentioned above, and some considerations are necessary. Fabric Q* represents the simplest composition, where the NPIs are essentially constituted by monocrystalline quartz with straight and undulose extinction of sedimentary and crystalline origin, respectively, as is also indicated by lithic fragments (Table 2 and Fig 9B). Mica grains are variably present, with muscovite prevalent on biotite. Hornblende and volcanic lithics are very rare. Among heavy minerals, epidote is quite frequent. These characteristics resemble the alluvial deposits of the Nile north of Khartoum, as well as the smectite-lllite clay fraction of the matrix. However, the limited presence of clay pellets and quartz and kaolinite aggregates points to the use of residual quartz sand of local origin for tempering the alluvial clay. As reported by several authors (e.g., [38, 42, 43]), the Nile mud between the 5^th^ and 6^th^ Cataract is dominated by the smectite-rich contribution of the Blue Nile. The bimodal texture of the fabric and the medium to low homogeneity of the paste represent further arguments for the use of tempering quartz.

The higher frequency of plutonic lithics and larger biotite crystals of fabrics QF* and KfsQ_m indicates that a crystalline source was nearby and offers clues for possible non-local origin. Fabric KfsQ_m has abundant coarse-sized and weathered alkali feldspars and biotite with few smaller plagioclases. Rare hornblende crystals were detected. The NPls’ roundness is generally low. The matrix consists of prevalent smectite-illite clay, like fabric Q*, but is more clayey. The texture is bimodal to seriate, and the mineralogical content of the NPIs is consistent with that of the matrix. The homogeneity (H) of the paste is medium-high (Table 2 and Fig 9C). The petrographic features point to the use of natural clayey sand, probably from colluvial deposits.

The samples of fabric QF* share a seriate texture with variable amounts of NPIs. In this case, quartz prevails over feldspars. Minor amounts of ilmenite grains are present in all QF* thin sections. As in KfsQ_m samples, biotite, and muscovite are common in the NPIs, and the matrix consists of smectite-illite clay (Table 2 and Fig 9A). The textural and compositional maturity is higher than that of fabric KfsQ_m.

The maximum grain sizes (D max) show quite good correlations with the archaeological periods, going from higher values in the Early Khartoum to lower ones in the Late Neolithic. Similar fabrics with frequent weathered feldspars (QF^#^_m) or Fe-oxides in the matrix (QF_i) were considered as variants. Also in these cases, natural clayey sand was prevalently used to prepare the ceramic paste since some samples are supposed to be tempered (Table 2).

**Fig 14:**
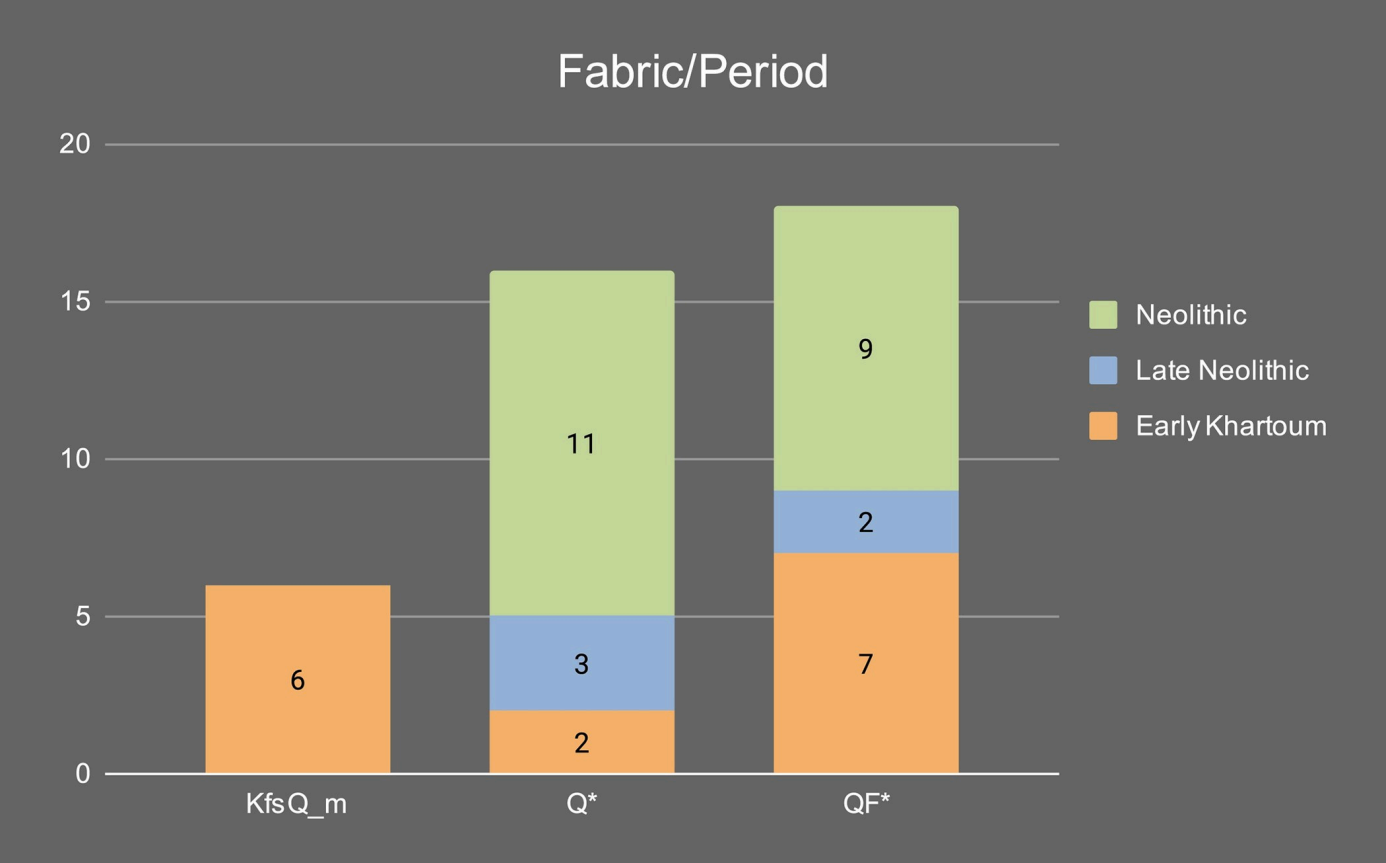
Distribution of the petrographic fabric groups in the different periods.

Of the three main fabrics, fabric KfsQ_m is exclusively associated with the earliest phase of occupation of the site in the Early Khartoum period (*n* = 6; 40% of the total Early Khartoum assemblage), whereas fabric QF* appears more diachronically distributed being in use from the Early Khartoum to the Neolithic and Late Neolithic periods. In particular, it most frequently occurs in the Early Khartoum (*n* = 7; 46.7% of the total Early Khartoum assemblage) and Neolithic (*n* = 9; 45.0% of the total Neolithic assemblage) but is also common in Late Neolithic (*n* = 2; 40.0% of the total Late Neolithic assemblage) (Fig 14). The provenance of this pottery may possibly be from several areas with a crystalline substratum. The proximity to the Nile and the number of settlements around Jebel Sabaloka make this area a possible candidate on the western bank of the Nile. The mica granite part of the ring-dyke, which bounds to the NW of the Cauldron Complex [7], supplies the colluvial sediments of this area and the petrofacies of the potsherds from Sphinx (on the west bank of Jebel Sabaloka) feature plutonic lithic fragments with characteristic micrographic texture and microperthitic microcline, although these last two features are not observed in our samples from Esh-Shaheinab [44].

South of Jebel Sabaloka, the Precambrian Basement (mainly metamorphic phases, i.e., gneiss and schist) outcrops up to the latitude of Esh-Shaheinab, if not further south, but is on the eastern bank of the Nile. Otherwise, younger intrusive phases (i.e., granitic-syenitic rocks) were available near the Early Khartoum sites of Saggai [45] and Kabbashi Haitah, south of Esh-Shaheinab, on the eastern Nile river bank [46]. In the Early Mesolithic pottery from Kabbashi Haitah, microcline-pertite, albitic plagioclase, quartz, biotite, and hornblende were considered weathering products of the local alkaline granites and syenite [46].

Finally, fabric Q* is associated with only 2 out of 15 Early Khartoum samples (13.3% of the total Early Khartoum assemblage), while the dominant correlation is with Neolithic samples (*n = 11*; 55.0% of the total Neolithic assemblage) and, secondarily, Late Neolithic samples (*n = 3*; 60.0% of the total Late Neolithic assemblage) (Fig 14).

Overall, the pottery from Esh-Shaheinab suggests a presumably long-lasting availability of certain clay raw materials, as well as a partial continuity of some technological traditions of raw material procurement throughout the entire timespan of the site. However, starting from the Neolithic period onward, a new trend in pottery making can be clearly noticed with an increased selection of ferruginous-based clays mainly tempered with quartz (fabric Q* and especially Q_i). Simultaneously, at the onset of the Neolithic period, the production of fabric KfsQ_m, which is characterized by a greater abundance of alkali feldspar and granite rocks, ends.

Further archaeometric data on prehistoric pottery in central Sudan are available. The petrographic analysis of the pottery from al-Khiday 17 km south of Khartoum, on the western bank of the White Nile, shows that alkali-feldspars and quartz-rich (Group 1) ceramic pastes and coarse quartz-rich ones (Group 2) are characteristic of Mesolithic potsherds, whereas the fine quartz bearing (Group 3) pottery is Neolithic [47]. Along the eastern bank of the Nile, petrographic data on the Early Mesolithic pottery from Kabbashi Haitah, 35 km north of Khartoum, show that ceramic pastes are dominated by alkali feldspar and quartz NPIs deriving from granitic-syenitic rocks cropping out in the environs of the site [46]. Earlier petrographic, mineralogical, and compositional studies conducted on the Mesolithic and Neolithic pottery of central Sudan confirm that the majority of the Early Khartoum ceramics (e.g., assemblages analyzed from sites of Saggai, Umm Marrahi, Sarourab, and El-Qoz, as well as the earliest ceramics from El-Kadada) were similarly composed of a suite of NPIs (i.e., K-feldspar, biotite mica, granite, and granitoid rocks) likely deriving from the petrofacies of the Precambrian Basement Complex. Differently, the Neolithic pastes are typically characterized by a suite of NPls (i.e., mainly quartz and Na-feldspar) and correlate with soils originating in regions where the Nubian Formation was exposed [27, 45, 48].

To summarize, POM and SEM-EDS analyses point to untempered clayey sand for the fabric KfsQ_m and tempered clays for Q*. Fabric QF* shows internal variability related to the different periods of the ceramic assemblages. The Early Khartoum and Late Neolithic samples appear to be untempered, unlike those from the Neolithic.

If the Q* fabric were likely made locally by tempering the Nile clay with residual quartz sand, the other two fabrics suggest non-local production, using colluvial clayey sands. The correlation of the petrographic data presented here with those from the area between Khartoum and Sabaloka finds more common elements between the fabrics KfsQ_m and QF* and the Precambrian Basement than the Sabaloka Ring Complex. However, a specific provenance cannot be identified without reference sediments.

### Manufacturing

#### Processing

The phase of processing of the paste and, in particular, the possible fractionation of the base clay or deliberate addition of tempering agents to improve the workability of the paste and the performance of the ceramic product is one of the most challenging aspects to evaluate within the entire manufacturing sequence [49]. For this reason, our petrographic analysis complemented the qualitative description of the compositional characteristics and main mineral phases of the ceramic fabrics by recording dedicated semi-quantitative parameters aimed at helping to discriminate paste transformation. Specifically, the degree of roundness of the NPls (R) expressed by ranges from 0–1 to 0–3, the presence of lithic fragments, the texture of the framework either unimodal (U), seriate (S) or bimodal (B), the degree of homogeneity of the paste (H), the presence of clay pellets (cp), and the primary porosity of the paste (P1) have been taken into account. In principle, a ceramic paste with compositional consistency between NPIs and matrix and high homogeneity of the paste does not appear to have been tempered. Otherwise, when the degree of homogeneity of the paste is low (0–1), the compositional difference between the NPIs and the matrix indicated by the presence of clay pellets, and a bimodal grain-size distribution occur, it is reasonable to consider a deliberate addition of tempering agents.

**Fig 15:**
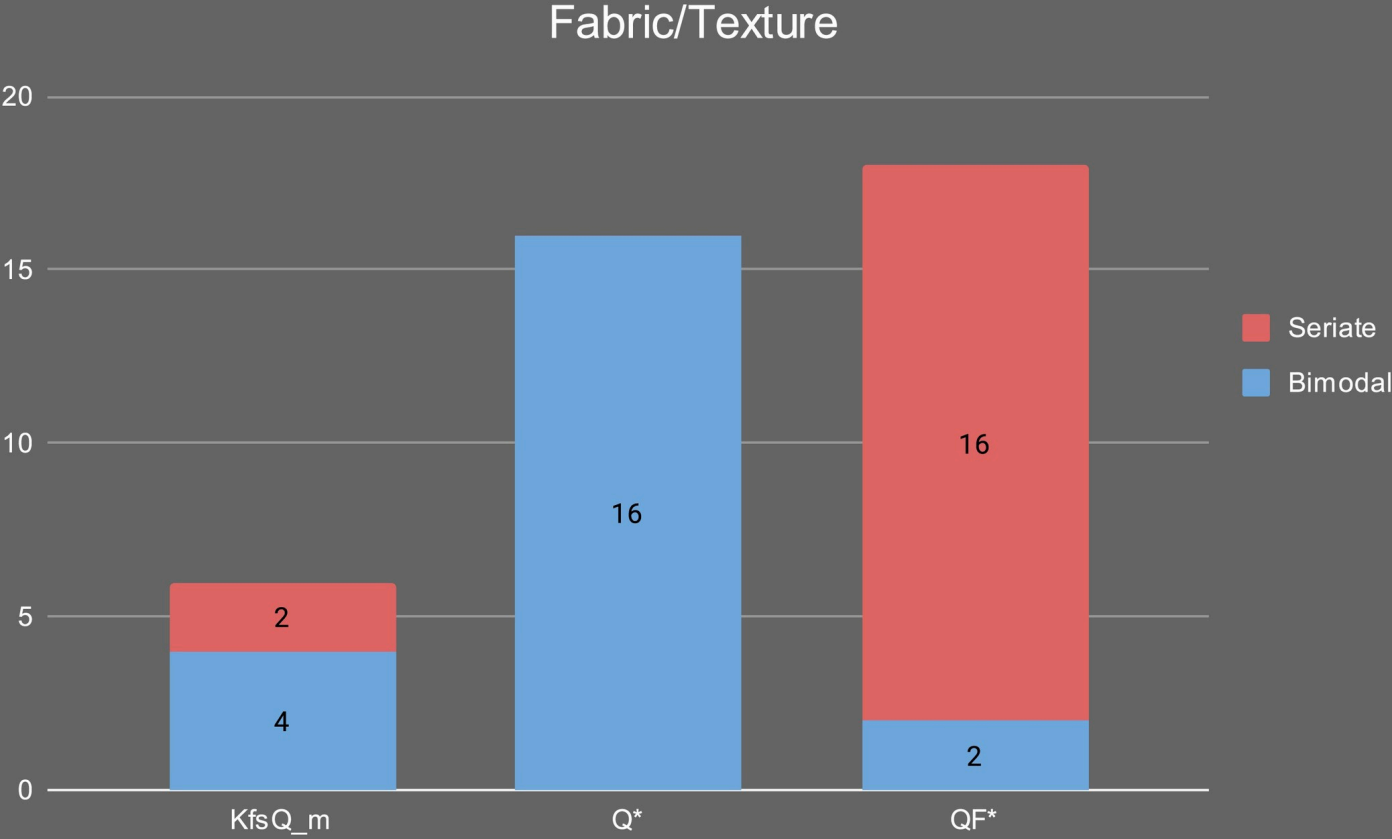
Distribution of seriate/bimodal textures in the different petrographic fabric groups.

In our case study, fabric KfsQ_m is characterized by a bimodal grain size on 4 out of 6 samples (Fig 15). Its primary porosity is medium to high, the paste normally has a low to medium degree of homogeneity, and the NPIs show a low degree of roundness. Samples with a seriate grain size largely dominate over bimodal ones (16 out of 18 samples) in fabric QF* (Fig 15), and pastes usually show a medium to high degree of homogeneity, medium degree of roundness, and medium primary porosity. Finally, all 16 samples of fabric Q* show a bimodal grain-size distribution, frequent clay pellets, and a low homogeneous distribution of NPIs (Fig 15).

The petrographic features of the first two fabrics point to a prevalent use of natural clayey sand to prepare ceramic pastes, with probable dry quarrying and subsequent hydration (clay pellets). The Early Khartoum samples of fabric KfsQ_m and the Early Khartoum and Neolithic samples of fabric QF* do not contain organic inclusions. However, all the fabrics show some carbonaceous matter due to the original presence of organic matter dispersed in the matrix.

Tempering of fabric Q* could be carried out during kneading and processing of the pastes by adding larger quartz inclusions to the base clay. Organic plant inclusions are also scanty in this fabric, which is primarily Neolithic. The SEM-EDS analysis demonstrated that no anomalous concentrations of P were detected in the paste, except for SHA050, where a weak Ca-phosphates contaminant occurs on the inner (lower) and outer surface (higher) (Fig 10F). The SEM-EDS analysis excluded the addition of herbivore dung to the clays and grog in all investigated samples. The high content of NPIs necessary to control the plasticity of the drying shrinkage of the paste consisting of a smectite-rich clay is antithetical to the need to add organic matter.

#### Paste preparation

The frequency of clay pellets (cp), the relative amount of primary porosity and shape (P1), and the homogenization of the paste (H) can be used as indicators of paste hydration and preparation. While P1 is widespread in all samples, fabrics Q_i and Q_m contain more clay pellets and a lower degree of homogeneity compared to fabrics KfsQ_m and QF*. Such differences can be attributed to incomplete hydration for Q* fabric and a general short kneading (Table 2).

#### Forming

**Fig 16:**
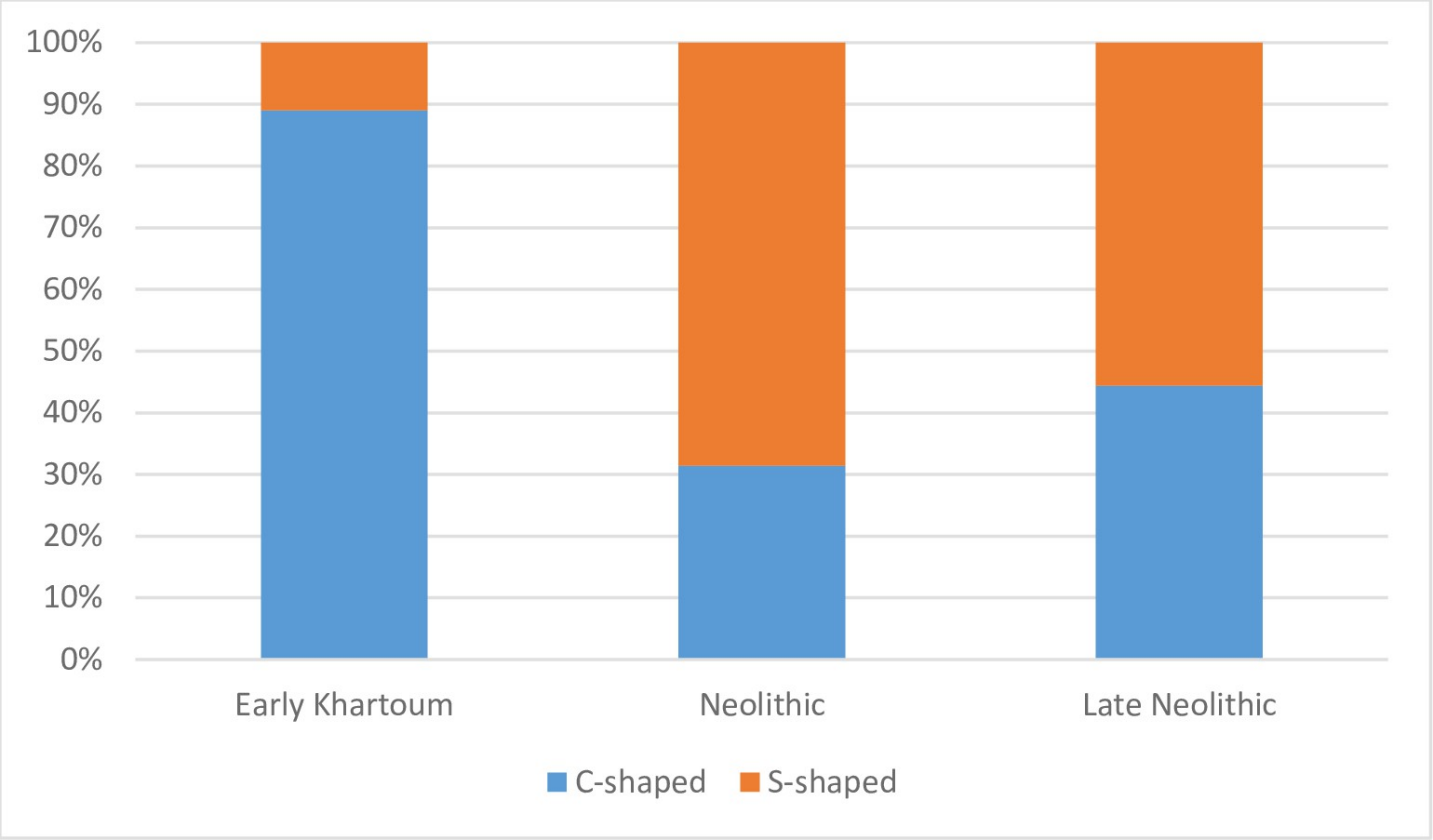
Distribution of coil shapes in the different periods.

**Fig 17:**
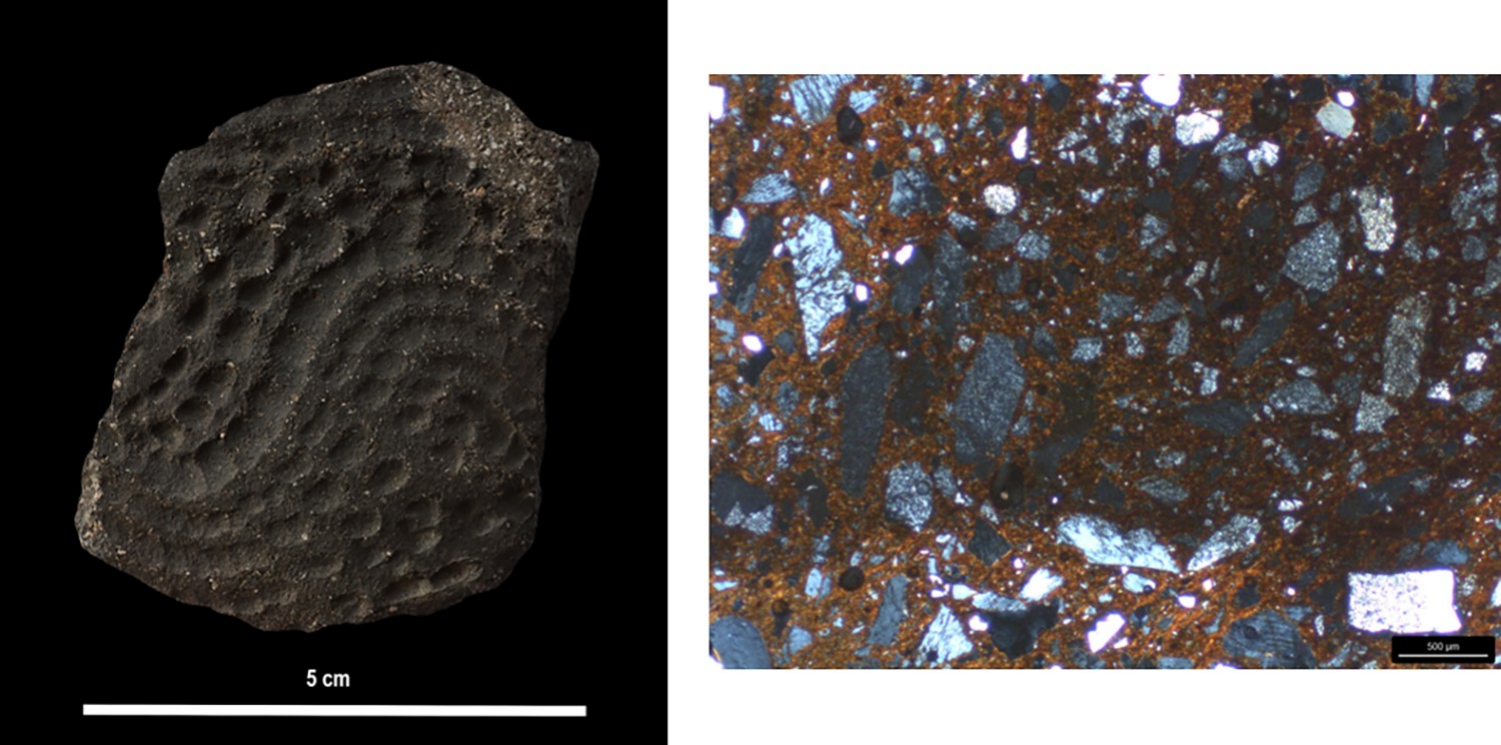
Coil shapes in cross-section (Photos by G. D’Ercole).

The absence or scarce isorientation of elongated NPIs and/or pores excludes forming actions involving rotative kinetic energy (RKE, after [50]). Macroscopic observations of the sherds’ cross section support the practice of superimposing coils. It was also possible to distinguish two types of coils, C-shaped coils, which exhibit a fairly circular diameter, and S-shaped coils, which have an oval cross-section. The former indicate that the coils have been superimposed without subsequent thinning, while the latter have been thinned by hand or by means of another tool. These two different practices are also time-sensitive. Early Khartoum pottery presents a prevalence of C-shaped coils (Fig 16), suggesting that it is predominantly made without thinning the walls, which is also confirmed by the greater vessel thicknesses. In contrast, Neolithic and Late Neolithic ceramics show a prevalence of S-shaped coils, indicating the practice of thinning the vessel walls (Fig 16). Coils are also visible in thin sections. They are highlighted by a circular arrangement of the mineral inclusions (Fig 17). As mentioned above in the macroscopic data, vessel shapes could only be reconstructed for Neolithic pots. They include a variety of sizes, with diameters spanning from a minimum of 7 cm to a maximum of 42 cm, and shapes, including ovoid, globular, everted, and straight-walled bowls and jars.

POM and SEM-EDS analyses confirm the macroscopic observations. The three fabrics do not show significant changes from the inner part toward the surface due to finishing of a still plastic body [51], although P2 is less frequent in the Q*fabric. A limited increase in compactness was observed in SHA897, which is undecorated (Fig 11C). Such a difference is consistent with the fact that a still plastic body was needed to make impressed and incised decorations, which occur on most specimens (Fig 8).

**Fig 18:**
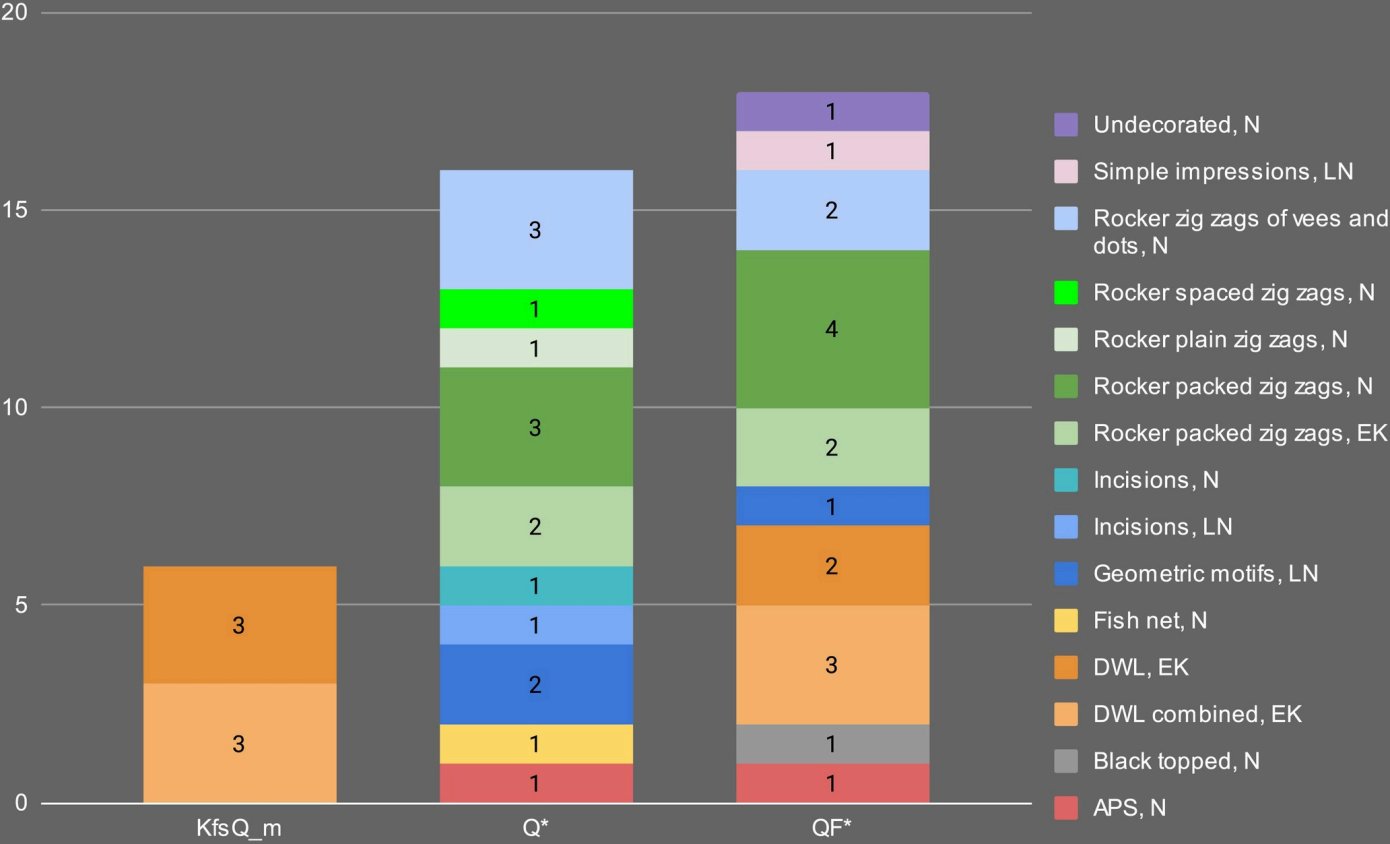
Distribution of decorative motifs in the different petrographic fabric groups (EK: Early Khartoum, N: Neolithic, LN: Late Neolithic).

Concerning the association between fabrics and decorations (Fig 18), fabric KfsQ_m appears to be exclusively associated with dotted wavy line motifs (both wavy banded and combined with zigzags) produced in the Early Khartoum period (Fig 8A). Conversely, fabric Q* mainly represents ceramics decorated with zigzag impressions, producing different motifs and dating to different periods (Fig 18). It includes packed zigzags made with evenly serrated tools, dating to the Early Khartoum (*n* = 2 samples) (Fig 8B) and the Neolithic (*n* = 3 samples) (Fig 8C) periods, as well as spaced zigzags (*n* = 1 sample), plain zigzags (*n* = 1 sample), and zigzags of vees and dots made with unevenly serrated combs (*n* = 3 samples) (Fig 8D), which are exclusively produced on burnished sherds in the Neolithic period. Finally, a minority of Neolithic and Late Neolithic samples are decorated with the incision technique and a few geometric patterns (Fig 8F), the latter dating to the Late Neolithic (*n* = 2 samples). One single Neolithic sherd of fabric Q_i is decorated with the alternately pivoting stamp technique. Furthermore, samples of fabric QF* are also mainly decorated with the rocker stamp technique, including both zigzag impressions (*n* = 8 samples) and Early Khartoum dotted wavy line motifs (*n* = 5 samples). Single specimens dated to the Neolithic and Late Neolithic periods show impressions made with the alternately pivoting stamp, simple impressions **(Fig 8E)**, black topped, and geometric motifs (Fig 18).

To sum up, fabric KfsQ_m is exclusively associated with Early Khartoum dotted wavy line motifs, but it cannot be ruled out that it could have been used to produce vessels decorated with other Early Khartoum motifs as well. In contrast, the other two fabrics (Q*and QF*) were used in all periods. However, Neolithic samples are predominantly composed of fabric Q*, while the amount of Neolithic samples of fabric QF* is equivalent to the sum of Early Khartoum and Late Neolithic samples.

Correlations between fabric groups and decorative types have also been noted in ceramic assemblages from other contemporary sites in central Sudan. K-feldspar and mica-rich fabrics are typically associated with incised wavy line and dotted wavy line decorations at the Early Khartoum site of Sphinx, on the west bank of Jebel Sabaloka (GDE, pers. observation). Furthermore, at Kabbashi Haitah, a strong connection between K-feldspar-rich pastes and wavy line motifs was observed, while ceramics decorated with the rocker impression are only found in association with a quartz-rich fabric [46]. At al-Khiday, on the White Nile, the rocker stamp pottery was also produced with pastes rich in K-feldspar tempers [47].

#### Firing

**Fig 19:**
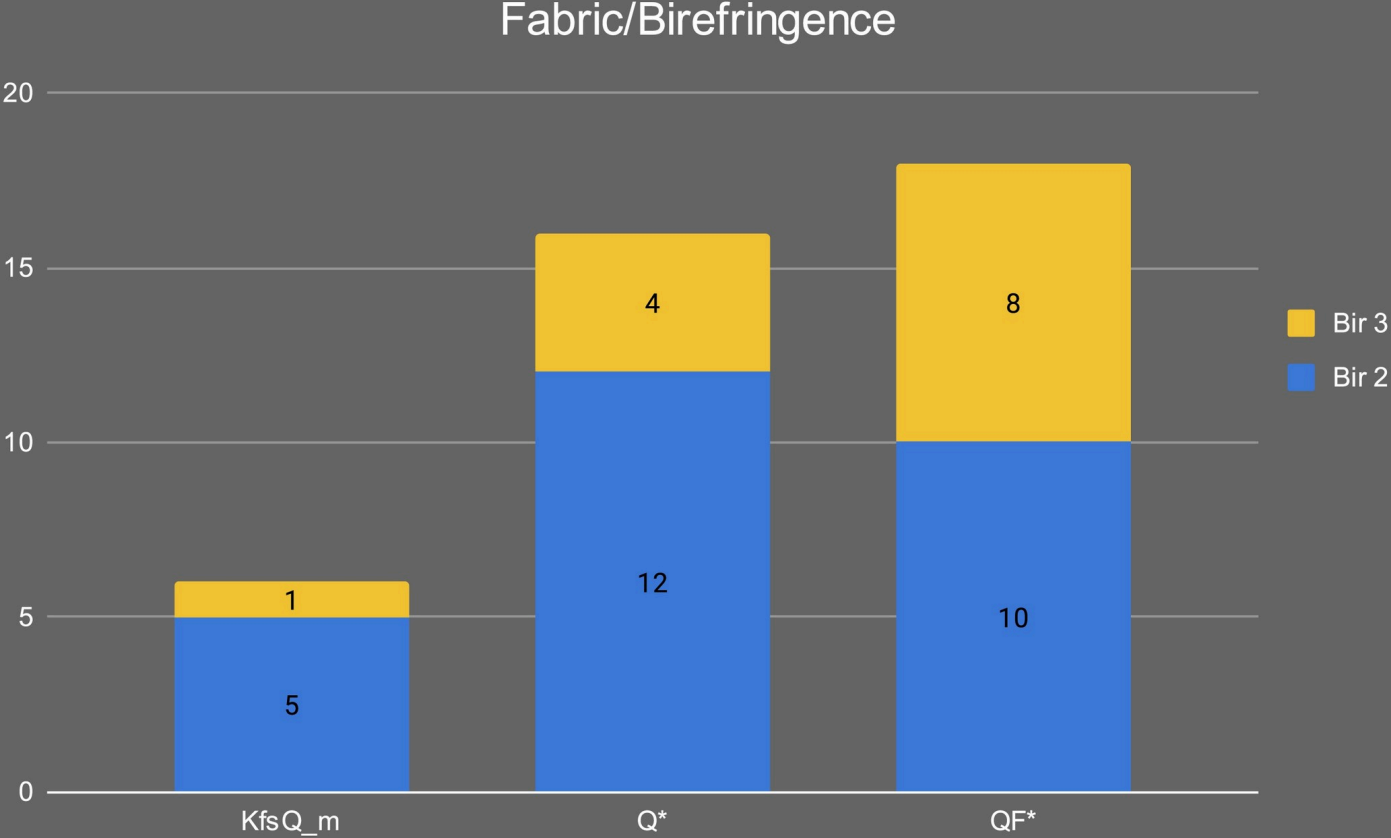
Distribution of birefringence classes (2: Medium; 3: High) in the different petrographic fabric groups.

**Fig 20:**
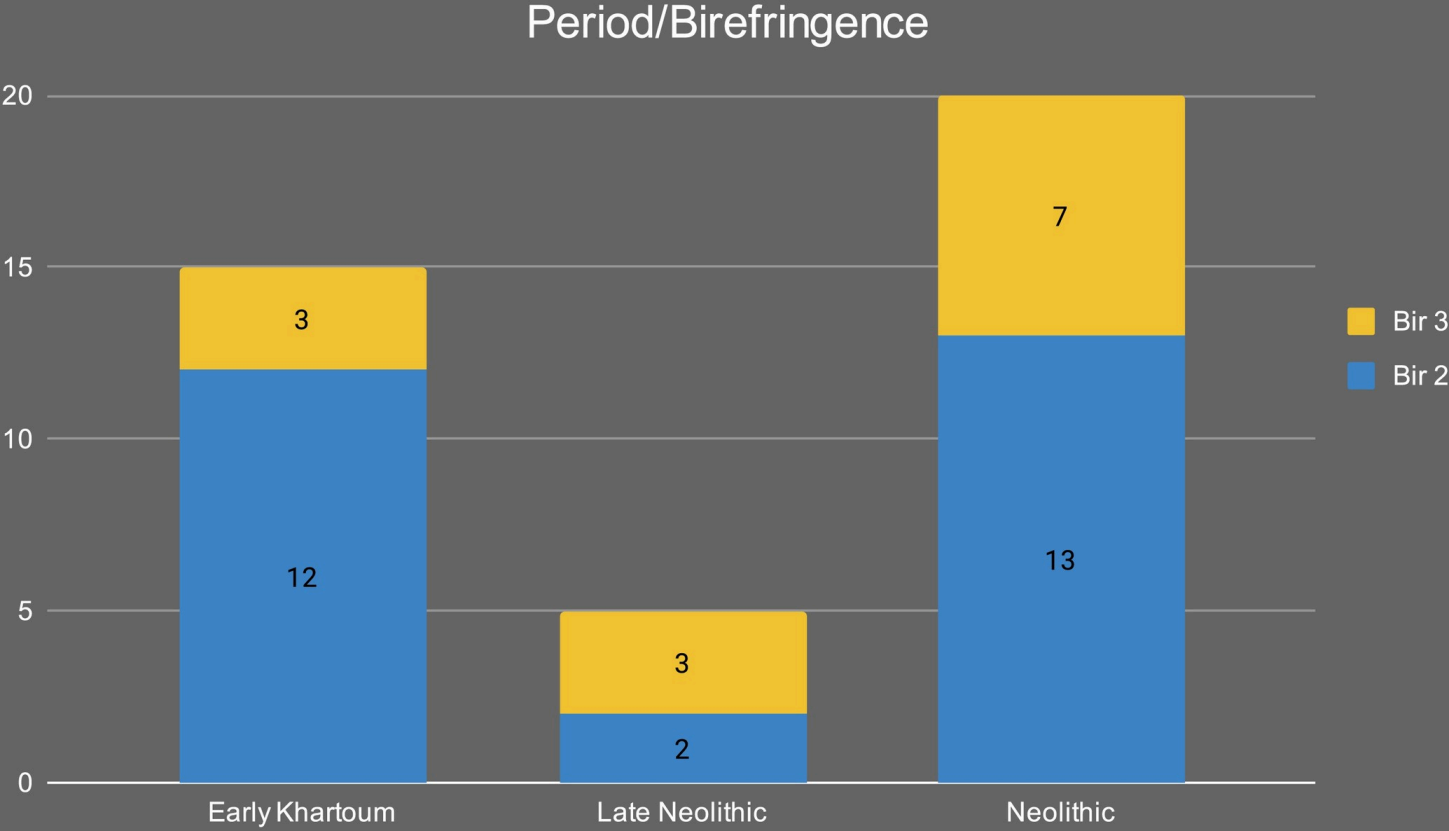
Distribution of birefringence classes (2: Medium; 3: High) in the Early Khartoum, Neolithic, and Late Neolithic periods.

Birefringence of the illite-rich matrix was classified between medium to high (2 to 3, Table 2). Incipient decrease of optical activity of the matrix at medium birefringence points to effects due to the first step of dehydroxylation (350–600°C) during firing [52, 53]. Since optical features of muscovite and biotite crystals of the NPIs are unaltered, equivalent firing temperature (EFT, after [54]) below 850°C [55] may be inferred. Compared to fabrics QF* and KfsQ_m, birefringence is relatively lower in fabric Q* and does not change in either the Early Khartoum, Neolithic, or Late Neolithic periods (Figs 19 and 20). EFTs below 600°C are probable for the vessels with a high birefringent matrix.

**Fig 21:**
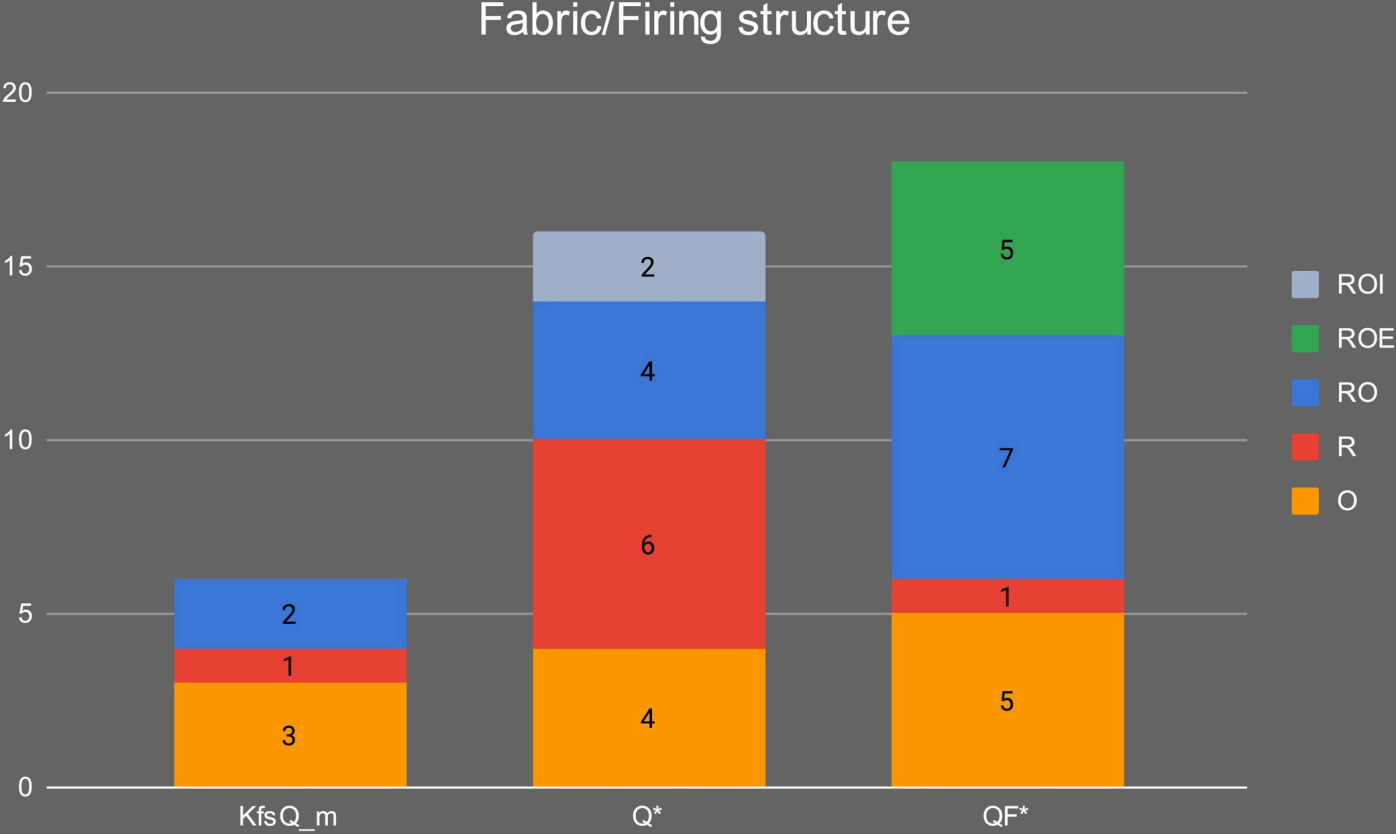
Distribution of firing structures in the different petrographic fabric groups (ROI: Reduced and oxidized on the inner surface; ROE: Reduced and oxidized on the external surface; RO: Reduced and oxidized; R: Non oxidized; O: Oxidized).

**Fig 22:**
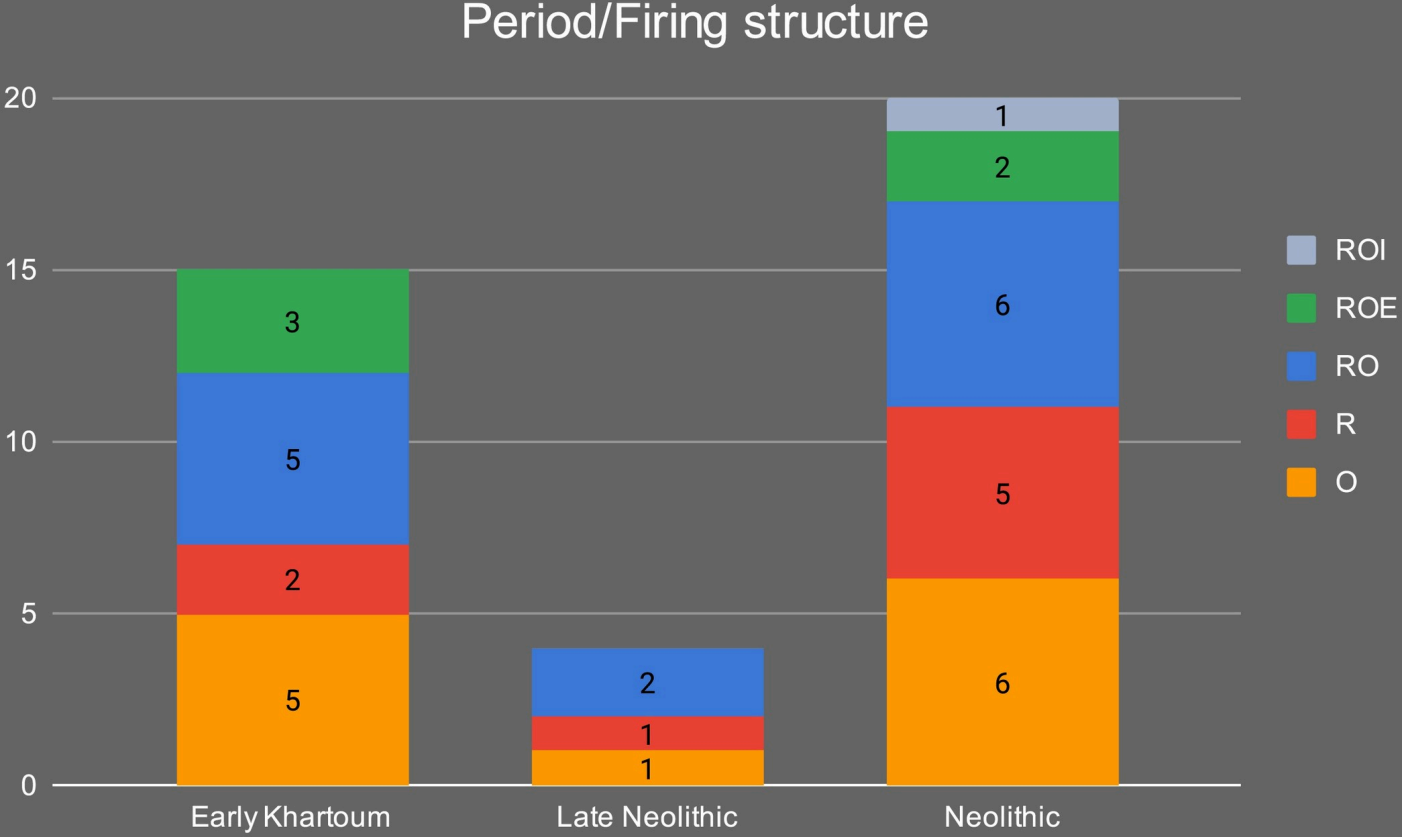
Distribution of firing structures among Early Khartoum, Neolithic, and Late Neolithic periods (ROI: Reduced and oxidized on the inner surface; ROE: Reduced and oxidized on the external surface; RO: Reduced and oxidized; R: Non oxidized; O: Oxidized).

A variability/asymmetry in the oxidation structures is observed in all the fabrics and periods, with RO (= a reduced domain followed by two symmetrical oxidized domains) being the most common combination pattern, followed by ROE (= reduced and then oxidized on the external surface), and ROI (= reduced and then oxidized on the inner surface) (see [56], 5). When the sequence of reduced and oxidized domains of the sample is ROI, it is likely that the pot was partially immersed in ash during cooling. On the other hand, when it is ROE, the vessel was supposedly fired upside down with short oxidation of the outer surface only occurring during the cooling phase. Oxidized (O) samples are represented in all fabric types. Non-oxidized (R) samples are especially common in fabric Q* and decrease in fabrics QF* and KfsQ_m. ROI structures only appear in combination with fabric Q*. Conversely, the ROE pattern seems to be a prerogative of fabric QF* (Fig 21). Overall, beginning with the Neolithic period onward, the number of vessels fired under only reducing (R) atmosphere increases, although other firing structures are still in use (Fig 22). Open or pit firing is compatible with incomplete oxidation of the matrix. The variable oxidation of the surfaces of the samples appears to be a consequence of direct contact with fuel.

### Vessel use

Whilst the lipid residue from the Early Khartoum period cannot, on its own, be used to interpret subsistence practices at that time, it is nonetheless interesting that the vessel was used to process non-ruminant carcass products, presumably from wild fauna such as the marsh cane rat (*Thryonomys swinderianus*) prevalent at that time. These can reach up to 10kg in size and have a higher protein but lower fat content than conventional later livestock, in good agreement with the low lipid content of this vessel (Table 4).The δ^13^C values from this vessel suggest that the animals producing these fats were subsisting on a wholly C_3_ diet, confirming what is known of the prevailing environmental (wetter) conditions present at this time (e.g. [57–59]). This is based on the principle that the foods animals eat display characteristic isotopic signatures [60] and thus isotopic analyses (δ^13^C) of fatty acids extracted from archaeological potsherds provide a reflection of the consumed diet, ultimately providing information about the environment in which the animals foraged [36, 61].

During the Neolithic, the processing of ruminant carcass products in vessels predominates (12 vessels, 44%, Fig 12), with a further 8 (30%) being used to process mixtures of ruminant and non-ruminant products. Some of this carcass processing likely originates from cattle (75%) and ovicaprids (25%), which make up 16% of the faunal assemblage. The wild ruminant fauna element of the assemblage (84%) comprises kudu, antelope, gazelle, giraffe, and hippopotamus [16]. The absence of plant lipid biomarkers in the Esh-Shaheinab sherds suggests that vessels plotting in the non-ruminant/plant range were used in the cooking of wild non-ruminants, likely turtle (mainly freshwater species), Nile monitor lizard (*Varanus niloticus*), Nile crocodile (*Crocodylus niloticus*), hare (*Lepus capensis*), and warthog (*Phacochoerus aethiopious*), all found at the site [16]. A further four Neolithic vessels (15%) were used in processing solely non-ruminant products, confirming the overwhelming dominance (89%) of the exploitation of meat at Esh-Shaheinab, whether through hunting of wild game or the management of domesticates. The very broad range of the δ^13^C values (from -26.0 to -15.2 ‰ for the δ^13^C_16:0_ fatty acids) during this time suggests the animals producing these fats consumed an extremely wide range of plant diets either composed mainly of C_3_ plants, varying combinations of C_3_ and C_4_ plants to a diet comprising primarily C_4_ plants, strongly confirming the exploitation of a broad range of animals.

It is worth noting that North Africa presents a unique scenario, where, unlike other continents, there is a large number of wild ruminants, including about 50–60 different species of bovids. Thus, the presence of ruminant adipose products cannot be regarded as a clear indication of the presence of domesticates and, as wild ruminant fauna are present in much greater abundance (84%) in the animal bone assemblage, the level of exploitation of domesticates at Esh-Shaheinab remains unclear, although the low number of domesticate remains and dairy residues (see discussion below) may hint that livestock were kept mainly for their secondary products.

Evidence for dairying is, however, an unambiguous indicator of domesticate exploitation. Although this seems to be a minor component at Esh-Shaheinab (7% of Neolithic and 12% of Late Neolithic vessels) the relatively high lipid concentration in vessel SHA880 at 0.9 mg g^-1^ (Table 4 and Fig 12A) suggests that at least this vessel saw sustained use, possibly in heating milk to make various milk products, such as cheese. Furthermore, all three vessels fall well within the dairy range, indicating that they were specialised for use in processing or storing milk/milk products. It should also be noted that the number of vessels used for dairy product processing may also be a reflection of container choice, for example, the use of organic containers (wood, ostrich eggshell, gourd, or leather) that do not survive in the archaeological record, rather than the degree of reliance on milk in the diet. Regardless, the use of vessels in processing dairy products is in good agreement with those at Neolithic al-Khiday [32], from at least 4500 cal BC, with two sherds (13%) yielding a ruminant dairy signal, very similar to results from the Neolithic phase (*c*. 4600–4000 BC) at Khor Shambat 1 where one vessel (5% of Neolithic assemblage) returned evidence for dairy processing [31]. Across a broader scale, the proportion of dairy vessels is consistent with recent results from the Nile Valley 8%, Wadi Howar 18%, Mediterranean North Africa 14%, Central Sahara 15%, and the Western Sahara 14% [62] However, these data are in contrast to the Early Neolithic site of Kadero (4600–3800 BC; [63]), where 47% of sherds yielded a ruminant dairy signal, which, together with livestock comprising 81% of the faunal assemblage, suggests that dairying was an important economic activity [29]. This may be due to the location of Kadero, which, although further from the Nile, has access to the larger expanses of alluvial plain on the east bank of the Nile, known to provide good grazing land [16]. Such varied results likely suggest local adaptations to specific ecological niches.

In the Late Neolithic, as noted, one vessel (12%) was used to process dairy products, with half of the vessels (*n* = 4, 50%) being used to process ruminant carcass products, likely reflecting the dietary practices of the nomadic pastoralists visiting the burial ground. Interestingly, as three vessels (38%) were used for cooking non-ruminant products, it seems likely that some exploitation of hunted non-ruminant wild game still occurred. There does not seem to be evidence of mixing of carcass products in these, however, it must be noted that this is a smaller dataset.

Similarly to other Nile sites, there is no evidence for the cooking or storage of fish in the Esh-Shaheinab pots, despite their remains being commonplace. This is in good agreement with a recent study of 359 vessels from sites across the mid to late Holocene Sahara [62] which strongly suggests that freshwater aquatic resources were not processed in ceramic vessels. Bearing in mind the presence of barbed bone harpoons and fishhooks at both Esh-Shaheinab and other sites along the Nile, the extensive availability of nearby surface waters and (sometimes) preponderance of fish fauna in deposits, for example, at al-Khiday fish remains averaged 90.5% of the 74,000 Mesolithic bones studied [64, 65], this seems somewhat strange. However, fish may have been processed by other means, for example, contemporary fish processing methods typically involve a combination of smoking, char-burning, deep-frying, and sun-drying. At Esh-Shaheinab, the absence of biomarkers denoting either plant or fish processing and the use of vessels to process purely animal products may suggest a deliberate cultural choice.

## Conclusions

Here, for the first time, we provide a multi-scalar analysis of the ceramic material from the renowned archetype site of Esh-Shaheinab, from the Early Khartoum to the Neolithic and Late Neolithic periods, by integrating the chaîne opératoire approach on ceramic assemblages within the landscape. Petrographic and mineralogical analyses of the composition of ceramic vessels and organic residue analysis (ORA) of the contents on the vessels have become recurrent in contemporary archaeological research. However, these two sets of analyses are rarely combined, even though their integration is a crucial means for reconstructing the entire operational sequence from raw material acquisition to production of the vessels up to their function and use. Furthermore, the integration of inorganic laboratory methodologies (i.e., POM and SEM-EDS analyses) and ORA provides a thorough understanding of past relationships and dynamics between human groups, their technological knowledge, cultural identity and traditions, and their surrounding cultural and environmental landscape.

In accordance with the environmental and archaeozoological data and with the organic residue analysis, petrographic and compositional results on the ceramic assemblages similarly disclosed a relatively stepwise transition from the Early Khartoum to the Neolithic and Late Neolithic periods, with the persistence of particular choices and traditions for the phases of raw material procurement and preparation of the ceramic pastes (i.e., feldspar-tempered fabrics and no evidence of herbivore dung as additive).

On the other hand, some discontinuities with replacement between the Early Khartoum and Neolithic productions of Esh-Shaheinab exist, especially regarding the phases of forming (S-shaped vs. C-shaped vessels), decoration (new variety of decorative techniques and motifs from the Neolithic on), and surface treatments (burnished vs. uncoated surfaces). Furthermore, fabric KfsQ_m, which is richer in alkali felspar and plutonic rock fragments and has a coarser texture than the other fabrics, was only produced during the Early Khartoum period. In parallel, the use of quartz-rich pastes (fabric Q*) became dominant starting from the Neolithic period onwards.

ORA results obtained from over 100 analysed sherds demonstrated the clear dominance of meat exploitation at the site, also during its Neolithic phase, with fatty acids δ^13^C values compatible with the exploitation of a broad range of animals, possibly including both domesticates and wild species. Earlier archaeozoological studies confirmed that domestic species only account for 16% at the Esh-Shaheinab Neolithic faunal remains and indicated a strong emphasis on wild meat consumption. This low frequency of domestic livestock may result from a gradual adoption of domesticates, exploitation for other purposes than meat, restricted killing, or environmental constraints of the western Nile bank [17].

Furthermore, evidence for dairying seems to occur to a lesser extent even during the Late Neolithic period, when half of the vessels were still being used to process ruminant carcass products and some exploitation of non-ruminant wild game still occurred. It is worth noting that these results differ from those produced from the Neolithic assemblage from the site of Kadero (where dairying was otherwise the main economic activity), however, this site is located in a different ecological niche, that is on the eastern bank of the Nile River.

Regarding the use of pottery and site economy, comparisons with the results of analyses on lipid residues of Neolithic vessels from other Sudanese sites, namely al-Khiday, Khor Shambat 1, and Wadi Howar, and other geographic regions, particularly Mediterranean North Africa, Central Sahara, and Western Sahara, indicate broad differences in resource exploitation systems even between contemporary sites. They suggest that local adaptations to specific ecological niches resulted in different dietary habits, spanning from a prevalent consumption of meat from wild or domestic ruminants to a notable consumption of dairy products or plant resources.

In addition to data on ceramic manufacturing techniques, this paper demonstrated that detailed analyses of ceramic assemblages are able to provide indications on a wider set of human behavior. The Esh-Shaheinab pottery mirrors the specific natural environment, settlement patterns, and available local resources of the inhabitants of this sector of the western Nile Valley, who had less extended alluvial plains and grazing land for their livestock in comparison to their “neighbors” on the opposite river bank. From a cultural perspective, the scenario observed at Esh-Shaheinab could also indicate a) a possible coexistence of foragers and herders for a certain time, and b) a certain degree of hybridization of pottery recipes and manufacturing processes between the Early Khartoum and Neolithic productions.

## Supporting information

S1 Table Faunal information. (XLSX)

S2 Table EDS Standards. (XLSX)

S3 Table EDS data used for producing the mean values of Table 3. (XLSX)

S1 Text Analytical methods. (DOCX)
