# Supplementary material for: Esh-Shaheinab: The archetype of the Sudanese Neolithic, its premises and sequels
Source: PLoS One. 2024 Oct 31;19(10):e0309600. doi: 10.1371/journal.pone.0309600 (PMC11527226; doi:10.1371/journal.pone.0309600)
Supplement: S1 Text — (DOCX) [file pone.0309600.s004.docx]

**Supporting information**

**Analytical methods**

***Polarized optical microscopy (POM)***

Thin sections for polarized optical microscopy (POM) were manufactured at the Institute of Geology of the Czech Academy of Sciences in Prague. Petrographic observations were performed by D’Ercole with a Zeiss Axio Lab.A1 polarized light microscope at the laboratories of the Department of Earth and Environmental Sciences - Section for Mineralogy, Petrology and Geochemistry of the Ludwig Maximilians-University in Munich and by Eramo with a polarising microscope Zeiss Axioskop 40 Pol at the Department of Earth and Geoenvironmental Sciences, University of Bari Aldo Moro, Italy.

Petrographic description considered non-plastic inclusions (NPIs), matrix and porosity. The boundary between matrix and NPIs is 15 μm according to Maggetti (1982). Beside the nature and percentage of NPIs, their grain-size distribution (i.e. texture, mode(s)) and homogeneity of distribution were considered. As for the matrix, the compositional features (e.g. calcareous, ferruginous and micaceous), the presence of clay pellets and the birefringence (e.g. high, medium and low) were noted. The oxidation structure of the matrix was classified according to the visual scheme proposed by Eramo and Mangone (2019). The acronyms refer to the sequence of reduced (R) or oxidised (O) domains of the fabric from the core outward. Asymmetric zoning is indicated by adding ‘I’ (internal) or ‘E’ (external) at the end of the acronym (ORI = Oxidised and then Reduced on the Inner surface). A ‘marbled’ (M) structure is characterised by patched reduced and oxidised domains. Regarding porosity, primary (i.e. pre-firing) and secondary (i.e. firing-induced) pores were distinguished under the microscope and their relative abundance estimated. Semi-quantitative estimation (% vol.) of NPIs and porosity was obtained by means of comparison charts (Matthew et al., 1991).

The fabric names described below provide information on the most abundant NPIs (prefix) and the nature of the matrix (suffix). As an example, the fabric name ‘QF_mo’ stands for prevalent quartz (Q) and feldspars (F) in a micaceous (m) and organic-rich (o) matrix. Other NPI abbreviations are as follows: A = argillaceous rock fragments (ARFs), Ka = carbonate aggregates, and Ve = vegetal inclusions. Further details on the methodology used are reported in Eramo and Mangone (2019).

***Scanning electron microscopy and energy dispersive spectroscopy (SEM-EDS)***

A selection of 9 thin sections (SHA025, SHA027, SHA034, SHA043, SHA044, SHA050, SHA053, SHA056, SHA897) were analyzed by SEM-EDS to verify the petrographic data obtained under the POM. Carbon coated samples were analyzed with a Zeiss Evo 50XVP Leo scanning electron microscope operated at 15 kV and 500 pA probe current. Energy dispersive spectrometric (EDS) microanalyses were conducted using an X-Max^N^ (80 mm^2^) SDD detector and AZtec software (Oxford Instruments) for X-ray maps and the correction of X-ray intensity was performed following Pouchou and Pichoir (1988, 1991). Microanalyses were run at about 25,000 cps output as average count rate on the whole spectrum, counting time 50 s and 8.5 mm as working distance. Different Micro-Analysis Consultants Ltd. (U.K.) mineral standards were used to check the accuracy of the analytical data. Relative errors ((|measured composition – certified composition|/certified composition) × 100%) of four of the standards used for element calibrations (augite, almandine, pyrope and orthoclase) are reported in the Standards EDS Table (S3 Table).

***Organic Residue Analysis (ORA)***

Lipid analysis and interpretations were performed using established protocols described in detail in earlier publications (Correa-Ascencio and Evershed 2014). Briefly, ~2 g of potsherd were sampled and surfaces cleaned with a modelling drill to remove exogenous lipids. The cleaned sherd powder was crushed in a solvent-washed mortar and pestle and weighed into a furnaced culture tube (I). An internal standard was added (20 µg *n*-tetratriacontane; Sigma Aldrich Company Ltd) together with 5 mL of H_2_SO_4_/MeOH 2 - 4% (δ^13^C value measured) and the culture tubes were placed on a heating block for 1 h at 70°C, mixing every 10 min. Once cooled, the methanolic acid was transferred to test tubes and centrifuged at 2500 rpm for 10 min. The supernatant was then decanted into another furnaced culture tube (II) and 2 mL of DCM extracted double distilled water was added. In order to recover any lipids not fully solubilised by the methanol solution, 2 x 3 mL of *n*-hexane was added to the extracted potsherds contained in the original culture tubes, mixed well and transferred to culture tube II. The extraction was transferred to a clean, furnaced 3.5 mL vial and blown down to dryness. Following this, 2 x 2 mL *n*-hexane was added directly to the H_2_SO_4_/MeOH solution in culture tube II and whirlimixed to extract the remaining residues, then transferred to the 3.5 mL vials and blown down until a full vial of *n*-hexane remained. Aliquots of the TLE’s were derivatised using 20 µl BSTFA, excess BSTFA was removed under nitrogen and the derivatised TLE was dissolved in *n*-hexane prior to GC, GC-MS and GC-C-IRMS. Firstly, the samples underwent high-temperature gas chromatography using a gas chromatograph (GC) fitted with a high temperature non-polar column (DB1-HT; 100% dimethylpolysiloxane, 15 m x 0·32 mm i.d., 0.1 μm film thickness). The carrier gas was helium and the temperature programme comprised a 50°C isothermal followed by an increase to 350°C at a rate of 10°C min^−1^ followed by a 10 min isothermal. A procedural blank (no sample) was prepared and analysed alongside every batch of samples. Further compound identification was accomplished using gas chromatography-mass spectrometry (GC-MS). FAMEs were then introduced by autosampler onto a GC-MS fitted with a non-polar column (100% dimethyl polysiloxane stationary phase; 60 m x 0.25 mm i.d., 0·1 μm film thickness). The instrument was a ThermoFinnigan single quadrupole TraceMS run in EI mode (electron energy 70 eV, scan time of 0·6 s). Samples were run in full scan mode (*m/z* 50–650) and the temperature programme comprised an isothermal hold at 50°C for 2 min, ramping to 300°C at 10°C min^-1^, followed by an isothermal hold at 300°C (15 min). Data acquisition and processing were carried out using the HP Chemstation software (Rev. C.01.07 (27), Agilent Technologies) and Xcalibur software (version 3.0). Peaks were identified on the basis of their mass spectra and gas chromatography (GC) retention times, by comparison with the NIST mass spectral library (version 2.0).

Carbon isotope analyses by GC-C-IRMS were also carried out using a GC Agilent Technologies 7890A coupled to an Isoprime 100 (EI, 70eV, three Faraday cup collectors *m/z* 44, 45 and 46) via an IsoprimeGC5 combustion interface with a CuO and silver wool reactor maintained at 850°C. Instrument accuracy was determined using an external FAME standard mixture (C_11_, C_13_, C_16_, C_21_ and C_23_) of known isotopic composition. Samples were run in duplicate and an average taken. The δ^13^C values are the ratios ^13^C/^12^C and expressed relative to the Vienna Pee Dee Belemnite, calibrated against a CO_2_ reference gas of known isotopic composition. Instrument error was ±0.3‰. Data processing was carried out using Ion Vantage software (version 1.6.1.0, IsoPrime).

**References**

Correa-Ascencio M, Evershed RP. High throughput screening of organic residues in archaeological potsherds using direct acidified methanol extraction. Analytical Methods 2014;6(5):1330-1340.

Eramo G, Mangone A. Archaeometry of ceramic materials. Physical Sciences Reviews 2019;4:331-356. <https://doi.org/10.1515/psr-2018-0014>.

Maggetti M. Phase analysis and its significance for technology and
origin. In: Franklin AD, Olin JS (eds) Archaeological ceramics.
Smithsonian Institution Press, Washington1982: 121-133

Matthew AJ, Woods AJ, Oliver C. Spots before the eyes: new
comparison charts for visual percentage estimation in archaeological
material. Recent developments in ceramic petrology 1991;81:211-263

Pouchou JL, Pichoir F. A simplified version of the “PAP” model
for matrix corrections in EPMA. In: Newbury DE
(eds) Microbeam Analysis. San Francisco Press 1988: 315-318.

Pouchou JL, Pichoir F. Quantitative analysis of homogeneous or
stratified microvolumes applying the model “PAP.” Electron probe
quantitation. Springer 1991; 31-75.
